# Unraveling transcriptomics of sorghum grain carotenoids: a step forward for biofortification

**DOI:** 10.1186/s12864-023-09323-3

**Published:** 2023-05-03

**Authors:** Clara Cruet-Burgos, Davina H. Rhodes

**Affiliations:** Department of Horticulture & Landscape Architecture, Colorado State University, Fort Collins, CO 80523 USA

**Keywords:** RNA-seq, Sorghum, Carotenoid, Biofortification, Vitamin A, Differential expression, Breeding

## Abstract

**Background:**

Sorghum (*Sorghum bicolor* [L.] Moench) is a promising target for pro-vitamin A biofortification as it is a global staple crop, particularly in regions where vitamin A deficiency is prevalent. As with most cereal grains, carotenoid concentrations are low in sorghum, and breeding could be a feasible strategy to increase pro-vitamin A carotenoids to biologically relevant concentrations. However, there are knowledge gaps in the biosynthesis and regulation of sorghum grain carotenoids, which can limit breeding effectiveness. The aim of this research was to gain an understanding of the transcriptional regulation of *a priori* candidate genes in carotenoid precursor, biosynthesis, and degradation pathways.

**Results:**

We used RNA sequencing of grain to compare the transcriptional profile of four sorghum accessions with contrasting carotenoid profiles through grain development. Most *a priori* candidate genes involved in the precursor MEP, carotenoid biosynthesis, and carotenoid degradation pathways were found to be differentially expressed between sorghum grain developmental stages. There was also differential expression of some of the *a priori* candidate genes between high and low carotenoid content groups at each developmental time point. Among these, we propose *geranyl geranyl pyrophosphate synthase* (*GGPPS)*, *phytoene synthase* (*PSY*), and *phytoene desaturase* (*PDS*) as promising targets for pro-vitamin A carotenoid biofortification efforts in sorghum grain.

**Conclusions:**

A deeper understanding of the controls underlying biosynthesis and degradation of sorghum grain carotenoids is needed to advance biofortification efforts. This study provides the first insights into the regulation of sorghum grain carotenoid biosynthesis and degradation, suggesting potential gene targets to prioritize for molecular breeding.

**Supplementary Information:**

The online version contains supplementary material available at 10.1186/s12864-023-09323-3.

## Background

Sorghum is a staple crop in many countries in South East Asia and Africa where vitamin A deficiency is a public health concern [1, 2]. Sorghum grain accumulates low concentrations of the pro-vitamin A carotenoids β-carotene, α-carotene, and β-cryptoxanthin, thus increasing their concentrations could improve the nutritional status of sorghum-consuming communities. Based on consumption patterns in Burkina Faso, Chad, Mali, Niger and Sudan — the five countries with the highest prevalence of vitamin A deficiency and consumption of sorghum — a biofortified sorghum would have to provide at least 2 µg/g to satisfy the nutritional needs of children under 5. These would represent an increase of about 2.5X of the sorghum lines with the highest known concentration of β-carotene [56]. Studies have demonstrated that sorghum carotenoids are controlled by genetic factors, suggesting that biofortification through breeding is possible [3–5]. However, understanding of the mechanisms underlying carotenoid biosynthesis and its regulation in sorghum grain is limited.

Carotenoid accumulation in sorghum is dependent on the developmental stage of the grain. A study of eight varieties of sorghum demonstrated that carotenoids accumulate in the grain differentially through development [6]. Carotenoid accumulation begins at around 10 days after half bloom (DAHB), reaches peak accumulation at 30 DAHB and then starts decreasing [6]. Similar differential accumulation of carotenoids through development has been observed for maize [7], tomato [8], and carrots [9–11]. Even though these studies each focus on different plant tissues—grain, fruit, and root, respectively—associations in each of the three crops have been identified between carotenoid accumulation through development and transcriptional differences [7–9, 11, 12]. Given that carotenoid biosynthesis is an essential pathway that is highly conserved in photosynthetic organisms [13–17], sorghum grain carotenoid accumulation through development could also be driven by transcriptional regulation. Alternatively, since the pattern of carotenoid accumulation in sorghum grain corresponds with the milky, soft dough, and hard dough developmental stages [18, 19], the differences in carotenoid accumulation could be a result of differences in nutrient mobilization, sequestration, storage, or plastid biogenesis. Understanding the mechanisms underlying carotenoid accumulation through development can help identify gene targets for breeding efforts.

Variation in carotenoid content in sorghum grain has also been identified among genotypes [3, 6, 20, 21], with yellow endosperm accessions accumulating higher concentrations. Genetic studies have found associations between carotenoid content and a few regions on the genome, indicating an oligogenic inheritance [3–5]. Many of the significant associations have been found near *a priori* candidate genes in the carotenoid biosynthesis (PSY, ZEP, and, LCYe) [3, 4], carotenoid degradation (CCD’s and NCED’s) [4], and the carotenoid precursor methylerythritol phosphate (MEP) pathways (MDS, GGPPS and DXR) [3], implying allelic variants in these genes are driving the variation in carotenoid content among sorghum genotypes. In maize, genomic studies have also suggested that grain carotenoids are oligogenic [22–27], and characterization of some of the allelic variants have shown differential gene expression among genotypes [7, 12, 24, 28–30]. However, some of the associated genomic regions could also be involved in other types of regulation, such as post-transcriptional regulation, affecting the enzymatic activities of these candidate genes. Knowledge of expression patterns of sorghum candidate genes in different genotypes could provide a better understanding of the mechanisms underlying carotenoid variation and help guide molecular breeding efforts by identifying which genes may be more impactful and should be prioritized.

Carotenoid regulation in plants is not fully understood, but it is hypothesized that they are regulated at multiple levels [31–33] because they are essential compounds for many key plant activities such as photosynthesis and photoprotection, as well as precursors to other important molecules such as abscisic acid (ABA). This implies that by direct or indirect regulation, many genes outside of the main carotenoid-related pathways must work in harmony to maintain adequate levels of carotenoids across multiple tissues, organs, and cellular compartments. Although most carotenoid studies in major crops have found evidence that carotenoids are oligogenic traits [3, 4, 22, 25–27, 34, 35], there is evidence that they have a polygenic architecture as well. The large number of quantitative trait loci (QTL) detected in these studies, as well as the low percentage of phenotypic variance explained by the QTL, suggest a more complex architecture. Furthermore, in maize, an RNA-seq experiment identified over 50 genes with expression associated with carotenoid concentration, among which only 19 of them were involved in carotenoid-related pathways [28]. Therefore, even though carotenoid biosynthesis and degradation in sorghum grain has been demonstrated to be controlled by only a handful of genomic regions, the total concentration observed in the grain could be dependent on many additive genetic factors.

A deeper understanding of carotenoid genetic architecture and regulation in sorghum grain will help to establish the best breeding schemes and selection methods for biofortification efforts. However, little is known about molecular mechanisms underlying sorghum carotenoid variation. We hypothesize that variation in carotenoid content is driven by variation in transcriptome profiles. To test this hypothesis, in this study we (1) identified which carotenoid *a priori* candidate genes are expressed in sorghum grain; (2) quantified transcriptional differences in carotenoid-related genes throughout sorghum grain development; and (3) characterized differences in transcriptional patterns among genotypes with contrasting carotenoid profiles. The results of this study can guide future breeding efforts for sorghum carotenoid biofortification by identifying genes underlying carotenoid variation at a transcriptional level.

## Results

### A priori candidate genes expressed in sorghum grain

To better understand the biosynthesis of carotenoids in sorghum grain, *a priori* candidate genes and their Phytozome-predicted (http://www.phytozome.net) transcripts were analyzed in order to test the hypothesis that known carotenoid-related genes are expressed in sorghum grain. The genes with transcript count numbers of 10 or more were categorized into three pathways: genes in the carotenoid precursor MEP pathway, genes in the carotenoid biosynthesis pathway, and genes in the carotenoid degradation pathways (Table 1, Additional File 2: Table S3). The carotenoid degradation pathways include genes involved in the biosynthesis of ABA and other apocarotenoids.

In the MEP pathway, all the *a priori* candidate genes were expressed in the grain (Table 1). For the carotenoid biosynthesis pathway, 19 out of the 20 genes identified as *a priori* candidates were expressed in the grain (Table 1). Sobic.010G276400, encoding a phytoene synthase (PSY), was not expressed; however, homologs of this PSY (Sobic.002G292600 and Sobic.008G180800) were expressed in the grain.

For the carotenoid degradation pathways, the *a priori* candidate gene list consisted of seven genes encoding carotenoid cleavage dioxygenases (CCD’s), involved in the conversion of β-carotene to apocarotenoids, and ten genes encoding 9-cis-epoxycarotenoid dioxygenases (NCED’s) or abscisic aldehyde oxidase (AAO), involved in the degradation of carotenoids to ABA. Five out of the eight CCD’s were expressed in the grain. The three CCD genes that were not expressed were Sobic.005G105700, Sobic.007G170300, and Sobic.010G050300. The ten genes encoding NCDE’s and AAO, were all expressed in the sorghum grain.

Differential transcript expression within some genes with multiple transcripts was observed. There were 3 out of the 22 MEP pathway genes, 6 out of the 20 biosynthesis genes, and 2 out of the 17 degradation genes that had had some transcript variants that were expressed and some variants that were not expressed. Differential transcript expression could underlie differences in carotenoid concentrations, however it is also possible that the transcript variants that were not expressed are not functionally important.

**Table 1:** A priori candidate genes expressed in sorghum grain

	Biochemical Pathway		
A priori candidate genes	Carotenoid Precursors (MEP Pathway)	Carotenoid Biosynthesis	Carotenoid Degradation
Expressed	22	19	14
Not Expressed	0	1	3
Total	22	20	17

### Factors controlling transcriptome variance

**Fig. 1:**
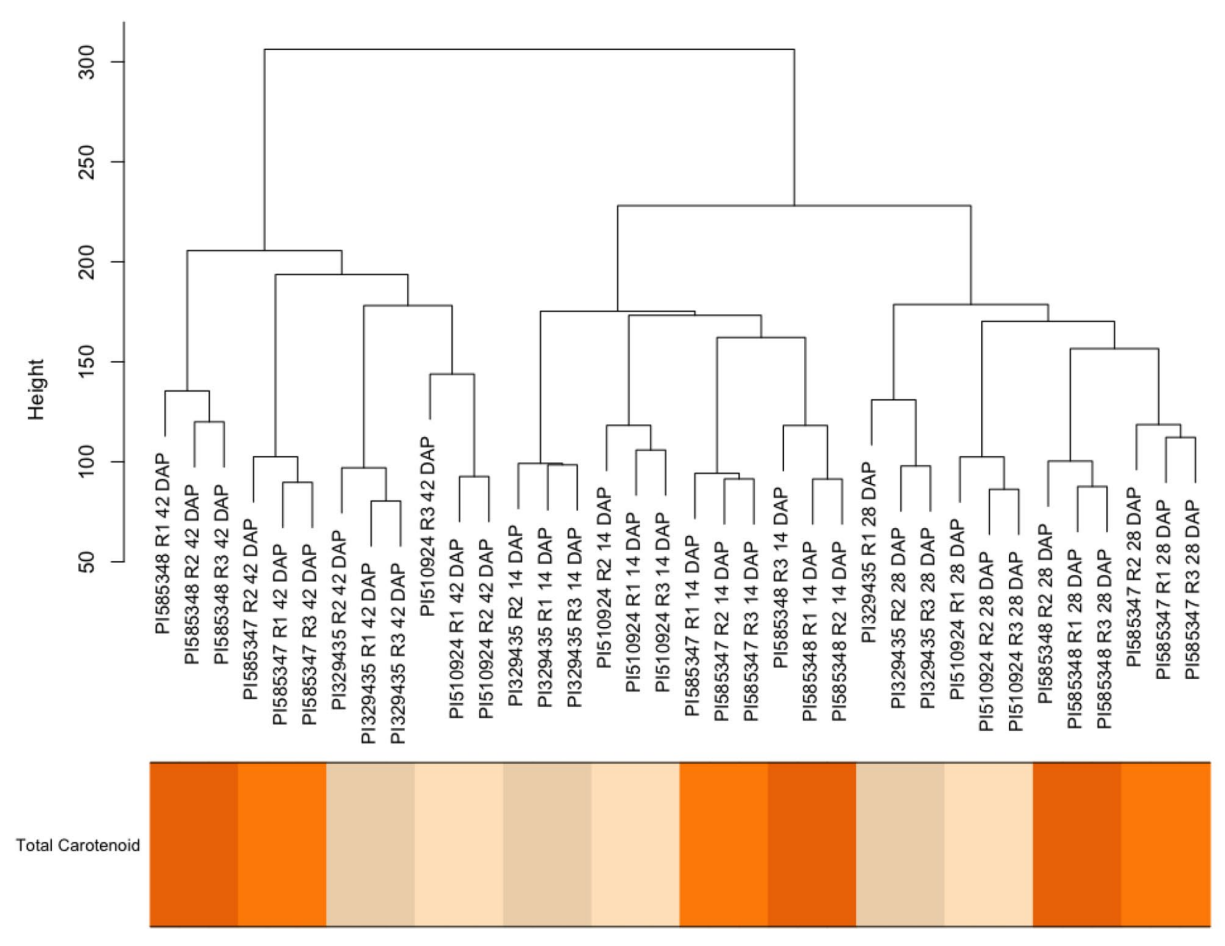
Samples hierarchical clustering and heat map of total carotenoid concentration at maturity for each accession. Darker colors in the heat map represent higher carotenoid concentrations, which were measured at maturity in a previous study [56]. R1-R3 denotes each biological replicate

Next, to generate hypotheses on patterns of expression, we explored the intergroup and intragroup (developmental time, high vs. low carotenoid lines, individual genotypes) transcriptomic variance for the samples. A hierarchical clustering analysis was conducted for the euclidean distance of the variance stabilizing transformation (VST) counts (Fig. 1). Developmental time, defined as 14, 28, or 42 days after pollination (DAP), was the factor that explained the majority of the transcriptomic variation, with perfect clustering of samples according to the DAP (Fig. 1). Within developmental time clusters, samples were grouped first by the carotenoid content group (High vs. Low), and then by individual genotypes (Fig. 1).

### Differentially expressed genes through grain development

Since developmental time point was the factor that explained the majority of the transcriptome variance, we next sought to test the hypothesis that carotenoid content differences through grain development are driven by differences in gene expression. A likelihood ratio test was performed to identify genes differentially expressed across grain development time points. A total of 21,236 transcripts were differentially expressed (False Discovery Rate (FDR) < 0.05) across grain development time points (Additional File 2: Table S4). Among them, 13 genes in the carotenoid precursor MEP pathway, 15 genes in the carotenoid biosynthesis pathway, and 13 genes in the carotenoid degradation pathway were differentially expressed across developmental time points (Additional File 2: Table S5, Additional File 2: Table S6).

**Fig. 2:**
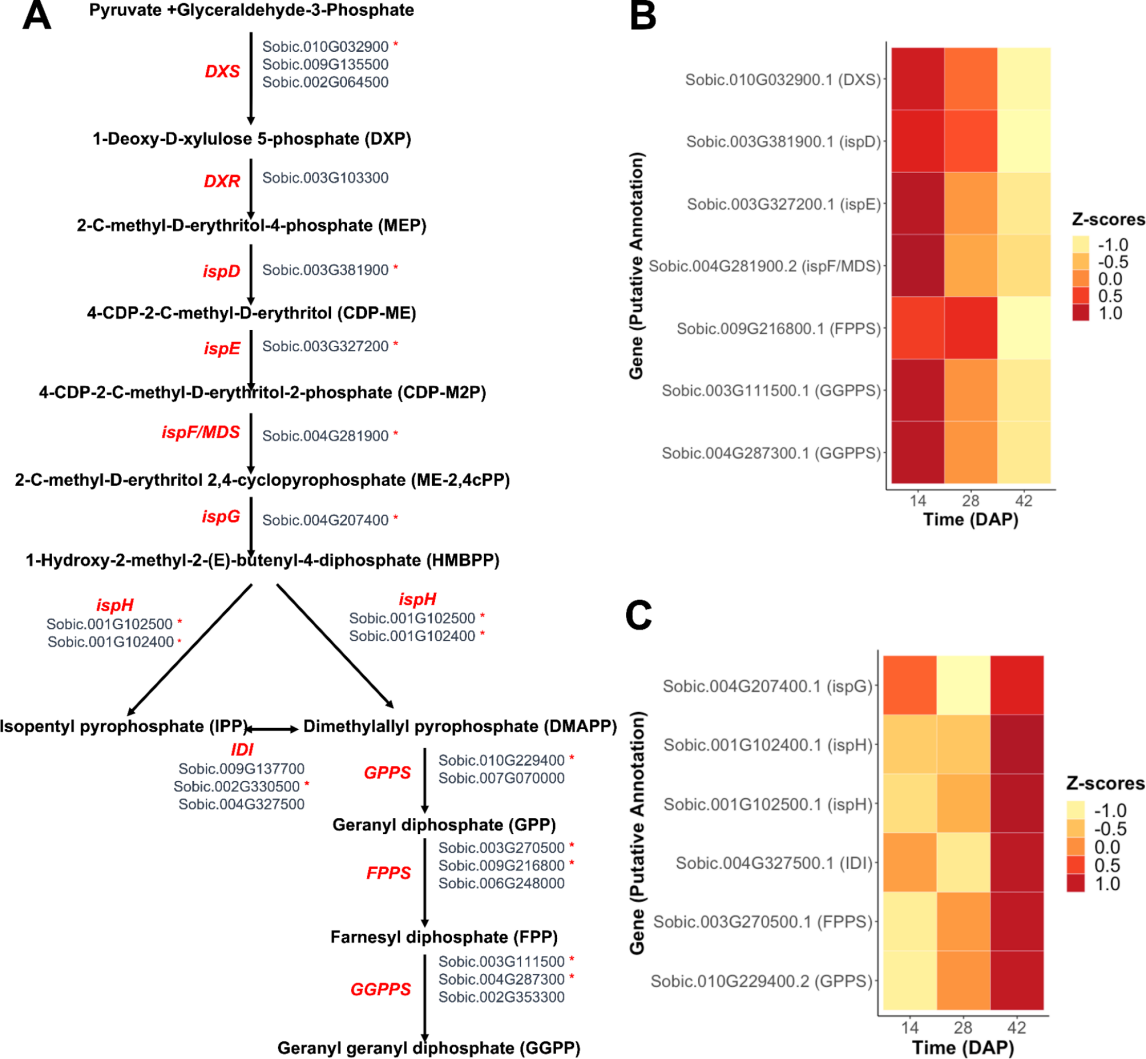
Precursor pathway: differentially expressed genes and patterns of expression across grain developmental time points (DAP). **A**) MEP pathway with the putative genes catalyzing each reaction. **B**) Genes expressed in a higher proportion at the beginning of grain development. **C**) Genes expressed in higher proportion at the end of grain development. Asterisks next to genes represent the genes that were differentially expressed across times. Positive Z-scores indicate higher expression compared to baseline

Most of the expressed genes in the MEP pathway were differentially expressed between grain development time points. The initial steps in the MEP pathway, up until the synthesis of 2-C-methyl-D-erythritol 2,4-cyclodiphosphate (ME-2,4cPP) (Fig. 2A), were more highly expressed during early stages of grain development (Fig. 2B). Interestingly, genes encoding the enzymes involved in the branching points for the production of isopentyl pyrophosphate (IPP) or dimethyl pyrophosphate (DMAPP) clustered in the opposite pattern with higher expression at the end of grain development (Fig. 2C). Aside from the genes involved in the branching points, only two other genes, Sobic.003G270500, encoding a farnesyl pyrophosphate synthase (FPPS), and Sobic.010G229400, encoding a geranyl diphosphate synthase (GPPS), were also in this cluster at the end of grain development (Fig. 2C). However, another *FPPS* (Sobic.009G216800) was highly expressed at initial stages of development (Fig. 2B), suggesting complementary function.

**Fig. 3:**
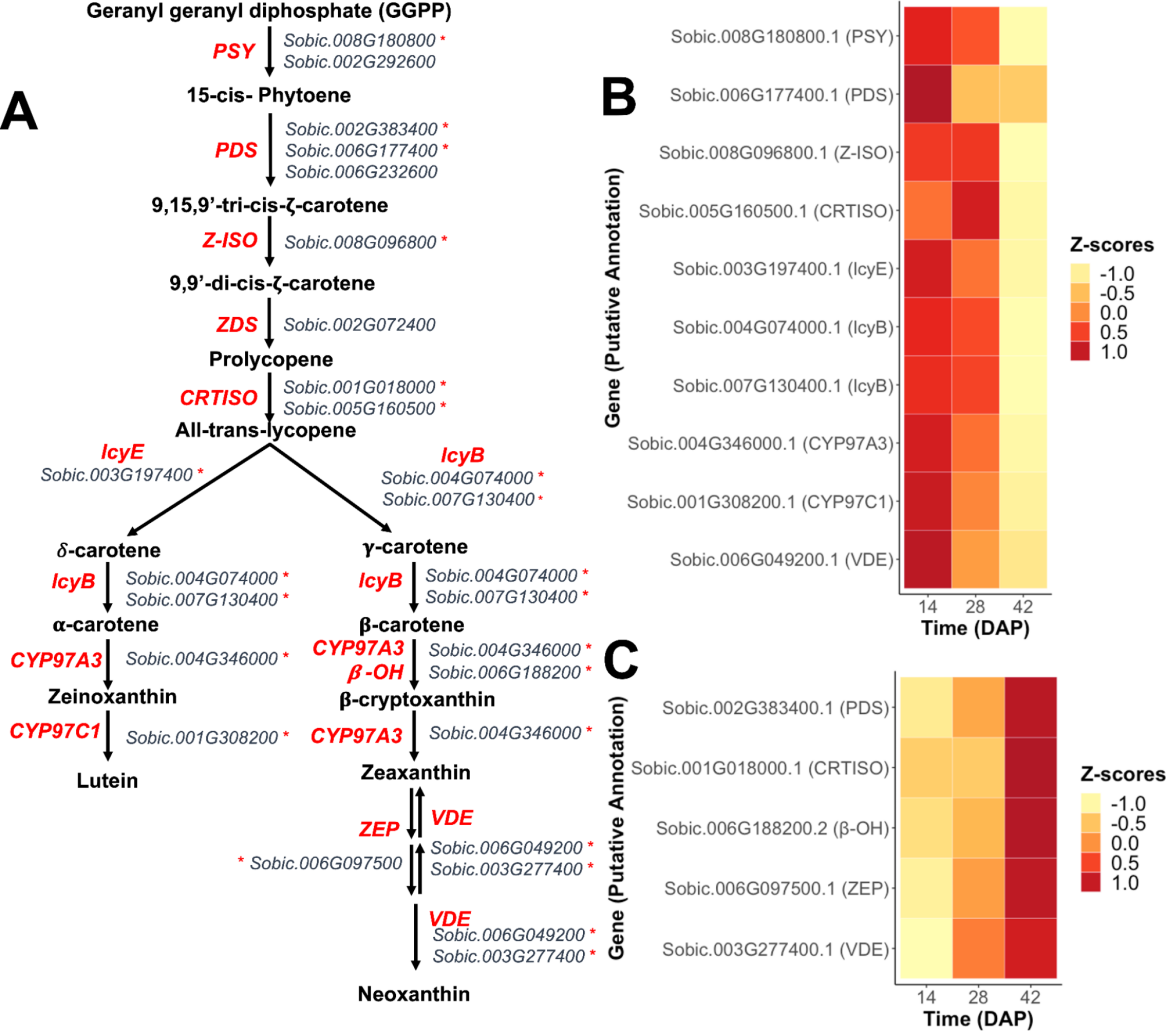
Biosynthesis pathway: differentially expressed genes and patterns of expression across grain developmental time points (DAP). **A**) Carotenoid biosynthesis pathway with the putative genes catalyzing each reaction. **B**) Genes expressed in a higher proportion at the beginning of grain development (14 DAP). **C**) Genes expressed in higher proportion at the end of grain development (42 DAP). Asterisks next to genes represent the genes that were differentially expressed across times. Positive Z-scores indicate higher expression compared to baseline

As expected, since previous studies show an increase in carotenoid concentration in early development [6], the expression of most carotenoid biosynthesis genes (Fig. 3A) was higher during early stages of grain development (Fig. 3B). Genes involved in the later steps of carotenoid biosynthesis, such as *β-carotene 3-hydroxylase* (*β-OH)*, *zeaxanthin epoxidase* (*ZEP)*, and *violaxanthin de-epoxidase* (*VDE)*, had the opposite trend with higher expression during later stages of grain development. Interestingly, there seemed to be complementations of expression for homologous genes encoding a carotenoid isomerase (CRTISO), phytoene desaturase (PDS) and VDE. For *CRTISO*, the Sobic.005G160500 homolog was more highly expressed during the early stages of grain development (Fig. 3B), whereas the Sobic.001G01800 homolog was more highly expressed during later stages of development (Fig. 3C). Similarly, the *PDS* homolog Sobic.006G177400 (Fig. 3B) was more highly expressed at 14 DAP, whereas the *PDS* homolog Sobic.002G383400 (Fig. 3C) was more highly expressed at 42 DAP. *VDE* homolog Sobic.006G049200 was more highly expressed during early stages of grain development (Fig. 3B), while Sobic.003G277400 was more highly expressed during later stages (Fig. 3C).

**Fig. 4:**
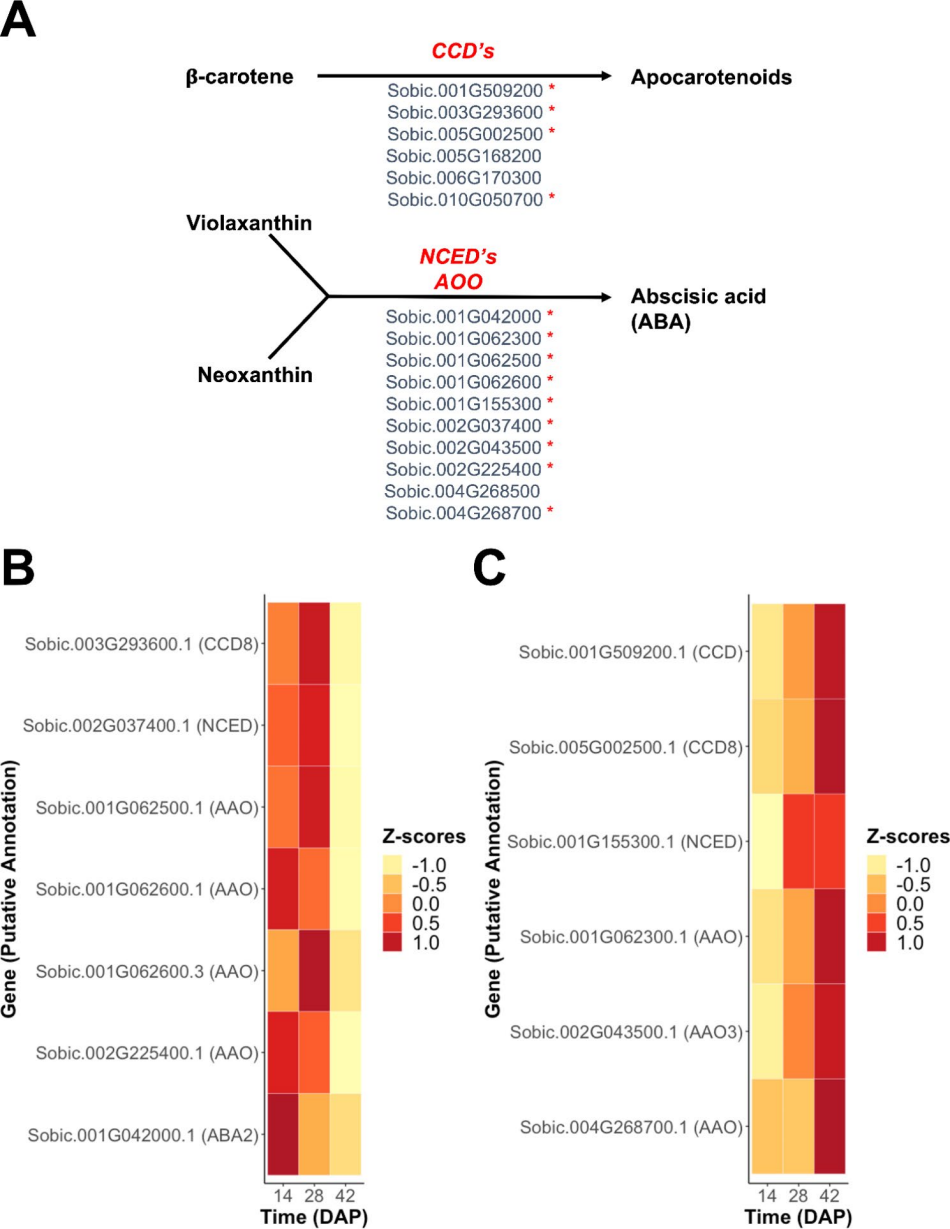
Degradation pathways: differentially expressed genes and patterns of expression across grain developmental time points (DAP). **A**) Carotenoid degradation pathways with the putative genes catalyzing each reaction. **B**) Genes expressed in a higher proportion at the beginning of grain development (14 DAP), **C**) Genes expressed in higher proportion at the end of grain development (42 DAP). Asterisks next to genes represent the genes that were differentially expressed across sorghum grain development time points. Positive Z-scores indicate higher expression compared to baseline

Carotenoid degradation genes (Fig. 4A) differentially expressed through grain development were also grouped into two clusters: highly expressed at early stages of grain development (Fig. 4B) and highly expressed at later stages of grain development (Fig. 4C). Genes encoding the three main enzyme types involved in carotenoid degradation—CCD’s, NCED’s, and AAO’s—were distributed in both clusters (Fig. 4B and 4C).

It is worth noting that a previous study characterized the differences in carotenoid accumulation through DAHB, which are days after half the plot has begun to flower under field conditions [6]. As our experiment was conducted in a greenhouse setting, we took flowering time (DAP) on a per plant basis. The correlation between DAHB and DAP can depend on the uniformity of flowering time of the accessions in the field.

### Differentially expressed genes between high vs. low carotenoid content groups

Next, since the carotenoid content group (high vs. low) was the factor that explained the majority of transcriptome variance after developmental time point, we sought to test the hypothesis that carotenoid content differences between high and low carotenoid lines are driven by differences in gene expression. The four sorghum accessions—PI329435, PI510924, PI585347, and PI585348—were chosen based on their low and high concentrations, respectively, as determined in a previous study [56] under field conditions (Additional File 2: Table S1). A Wald test was conducted to identify genes differentially expressed between the high and low carotenoid accessions for each of the developmental time points and the VST transformed transcript counts were compared for differentially expressed genes between accessions.

**Fig. 5:**
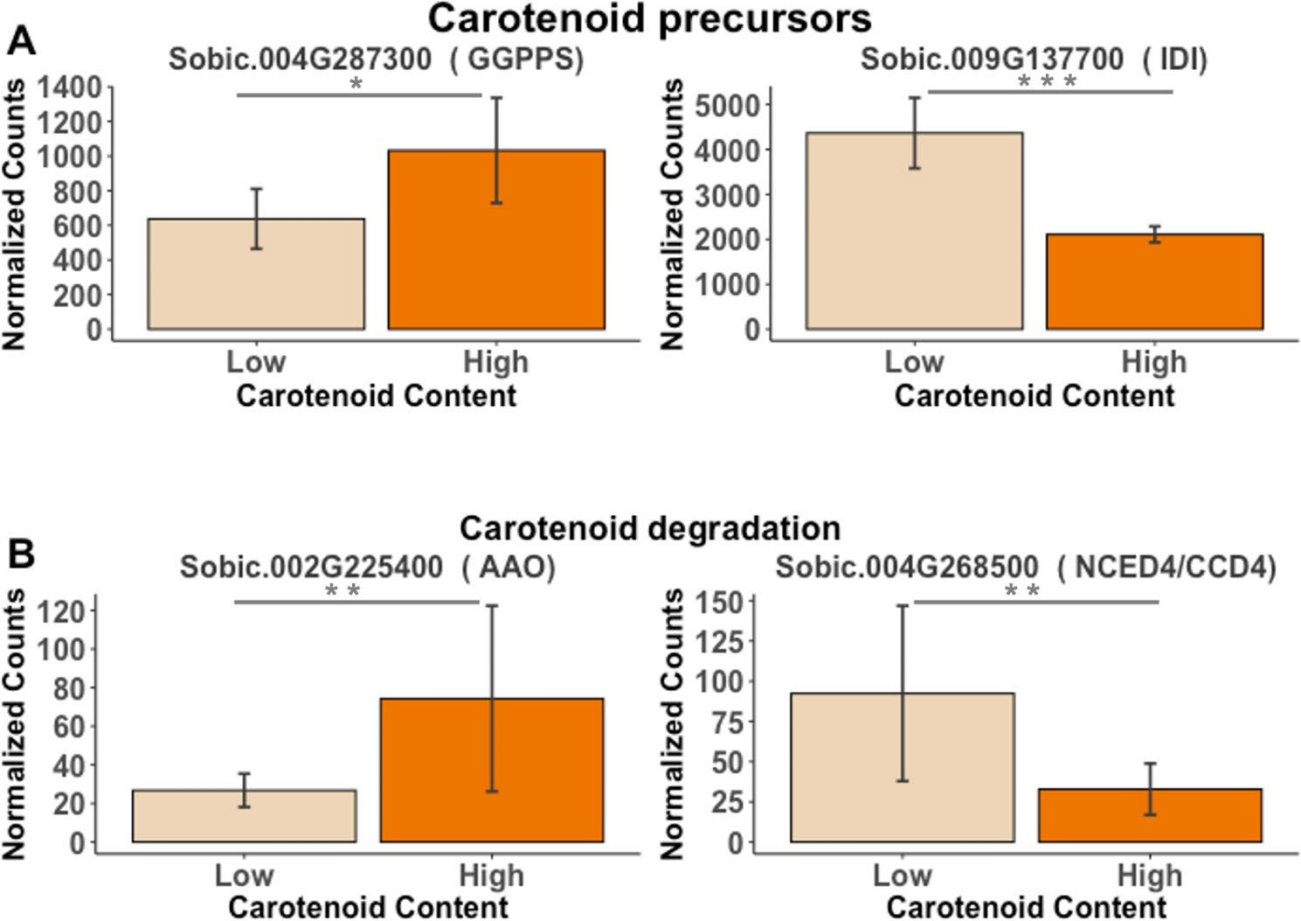
Normalized counts for differentially expressed genes between high and low carotenoid accessions at 14 DAP. **A**) carotenoid precursors (MEP pathway); **B**) carotenoid degradation pathways. PI329435 and PI510924 represent the low carotenoid group, while PI585347 and PI585348 represent the high carotenoid group. Wald test p-value is represented by ‘***’, ‘**’ and ‘*’ for 0.001, 0.01 and 0.05, respectively

A total of 2,587 genes (2,593 transcripts) were differentially expressed between high and low carotenoid content lines at 14 DAP (Additional File 2: Table S7). However, among these genes none were *a priori* candidates in the carotenoid biosynthesis pathway, and only four of them were *a priori* candidate genes involved in either the carotenoid degradation or the carotenoid precursor MEP pathway (Fig. 5, Additional File 1: Fig. S1). In the MEP pathway, Sobic.004G287300 (*GGPPS*) and Sobic.009G137700 (*IDI*) were differentially expressed between high and low carotenoid lines. The *GGPPS* was more highly expressed in the high carotenoid content group, whereas the *IDI* was more highly expressed in the low carotenoid group (Fig. 5A, Additional File 2: Table S8). These results could suggest that there might be a feed-forward regulation in the MEP pathway resulting in higher carotenoid content. In the carotenoid degradation pathways, Sobic.004G268500 (*NCED4/CCD4*) and Sobic.002G225400 (*AAO*) were differentially expressed between high and low carotenoid lines. However, the expression of these two genes was relatively low. The *NCED4/CCD4* was more highly expressed in the low carotenoid group, whereas the *AAO* was more highly expressed in the high carotenoid group (Fig. 5B, Additional File 2: Table S8).

**Fig. 6:**
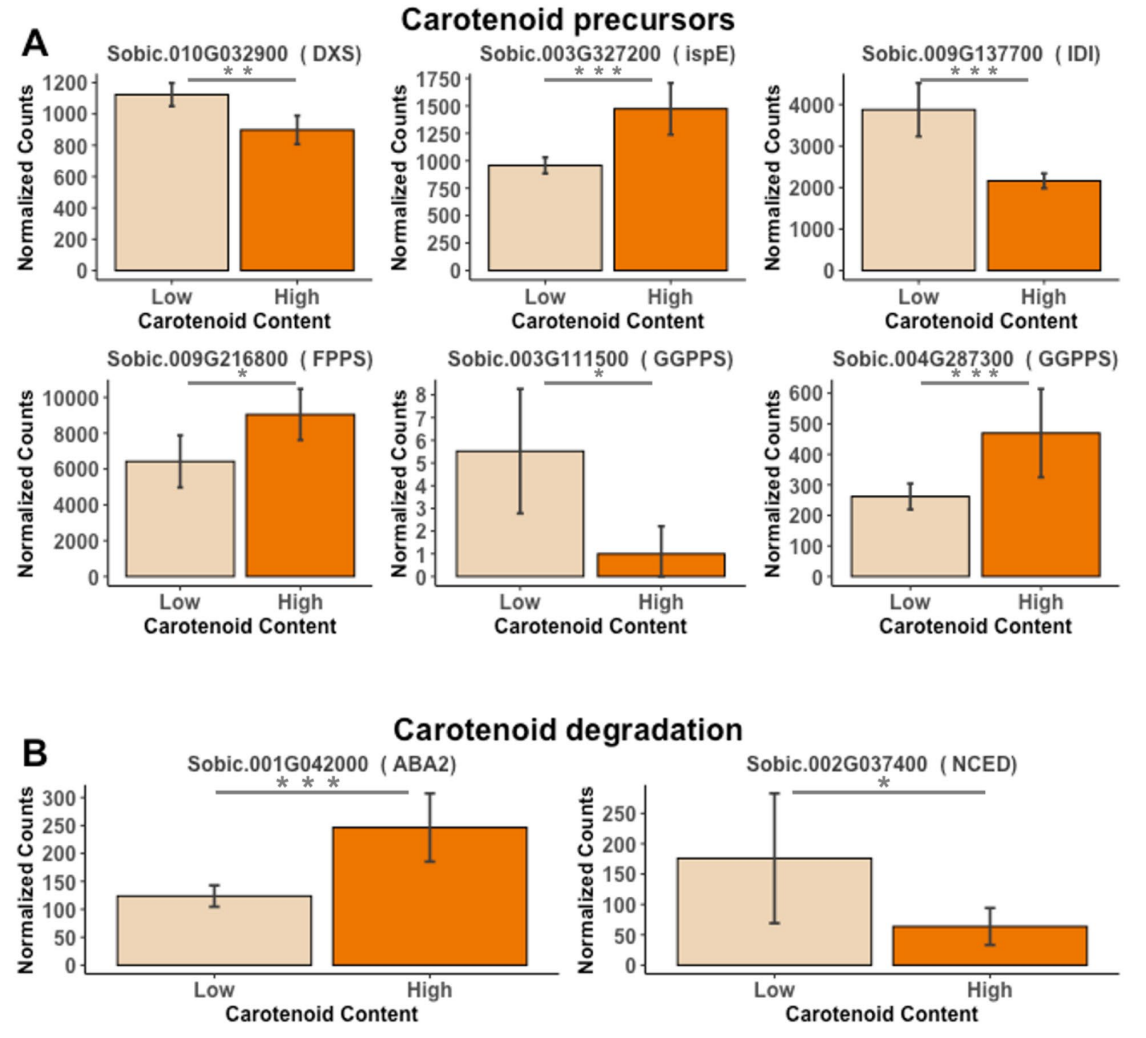
Normalized counts for differentially expressed genes between high and low carotenoid accessions at 28 DAP. **A**) carotenoid precursors (MEP pathway); **B**) carotenoid degradation pathways. PI329435 and PI510924 represent the low carotenoid group, while PI585347 and PI585348 represent the high carotenoid group. Wald test p-value is represented by ‘***’, ‘**’ and ‘*’ for 0.001, 0.01 and 0.05, respectively

The highest total number of differentially expressed genes for the carotenoid content group (high vs. low) was found at 28 DAP with 4,919 genes (4,940 transcripts) (Additional File 2: Table S9). Eight genes were *a priori* candidate genes with two of them in the carotenoid degradation pathways and the remaining six in the MEP pathway (Fig. 6, Additional File 1: Fig. S2). Genes differentially expressed in the MEP pathway for the high vs. low carotenoid content groups had different patterns (Fig. 6, Additional File 2: Table S10). Of the two GGPPS genes that were differentially expressed, Sobic.003G111500 had higher transcript counts in the low carotenoid group compared to the high carotenoid group, but it had a very low transcript count overall, which could suggest it is not the main GGPPS enzyme functioning in the grain (Fig. 6A, Additional File 2: Table S10). Two genes, Sobic.010G032900 (*deoxyxylulose 5-phosphate synthase*, *DXS*) and Sobic.009G137700 (*IDI*) had higher expression in the low carotenoid content group. The *IDI* gene was also identified as differentially expressed among the carotenoid content group for 14 days and had the same pattern of expression with higher expression in the lower carotenoid content group. The remaining three genes, Sobic.003G327200 (*ispE*), Sobic.009G216800 (*FPPS*), and Sobic.004G287300 (*GGPPS*), were more highly expressed in the high carotenoid lines at 28 DAP (Fig. 6A). For carotenoid degradation, an *NCED* (Sobic.002G037400) and an *ABA2* (Sobic.001G04200) were differentially expressed (Fig. 6B). Similar to 14 DAP, these carotenoid degradation genes had opposite patterns of expression with the *NCED* more highly expressed in the low carotenoid group.

**Fig. 7:**
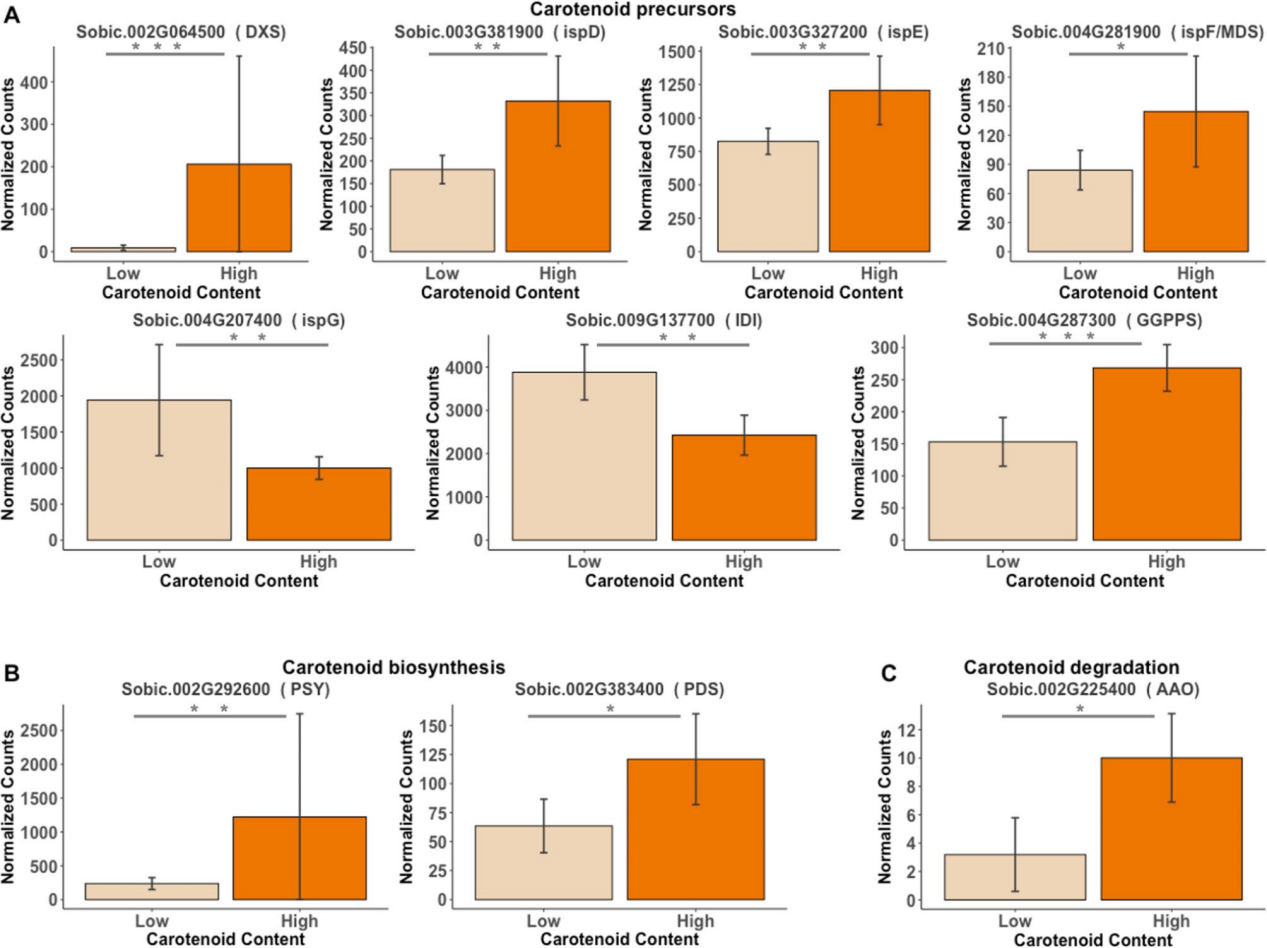
Normalized counts for differentially expressed genes between high and low carotenoid accessions at 42 DAP. **A**) carotenoid precursors (MEP pathway); **B**) carotenoid biosynthesis pathway; C) Carotenoid degradation pathways. PI329435 and PI510924 represent the low carotenoid group, while PI585347 and PI585348 represent the high carotenoid group. Wald test p-value is represented by ‘***’, ‘**’ and ‘*’ for 0.001, 0.01 and 0.05, respectively

At 42 DAP, there were 4,294 genes (4,327 transcripts) differentially expressed among the carotenoid content group, which included the highest number of *a priori* candidate genes (Fig. 7, Additional File 1: Fig. S3, Additional File 2: Table S11). Compared to the carotenoid biosynthesis and degradation pathways at this time point, the precursor MEP pathway had the highest number of genes differentially expressed between high and low carotenoid lines (Fig. 7A). Among them, three genes were differentially expressed with the same pattern of expression at previous time points: Sobic.004G287300 (*GGPPS*) and Sobic.009G137700 (*IDI*) at both 14 DAP and 28 DAP, and Sobic.003G327200 (*ispE*) at 14 days.

**Fig. 8:**
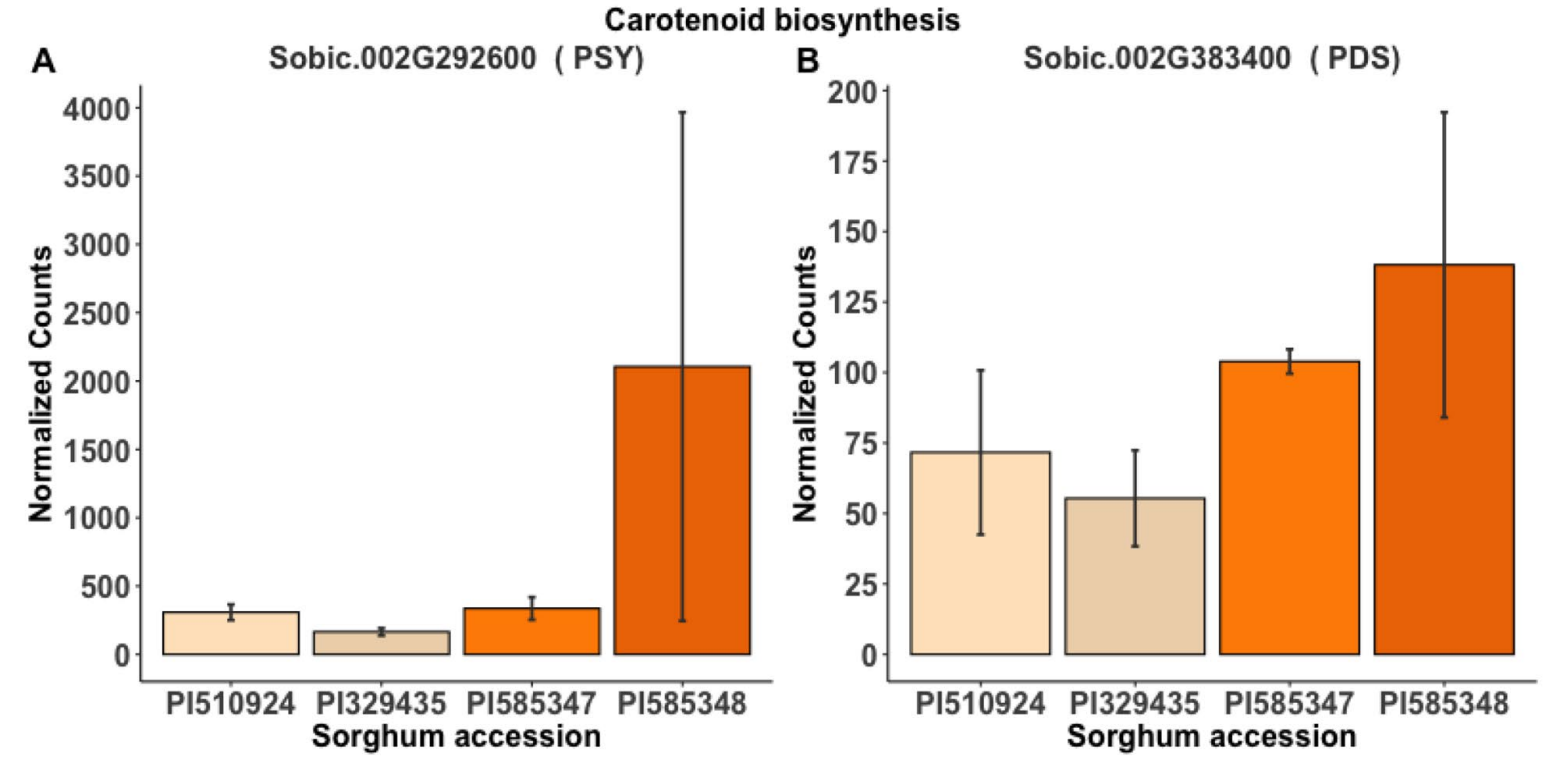
Genotypes normalized transcript counts for carotenoid biosynthesis genes differentially expressed at 42 DAP in high versus low carotenoid sorghum accessions. **A**) Phytoene Synthase (PSY); **B**) Phytoene desaturase (PDS). PI329435 and PI510924 represent the low carotenoid group, while PI585347 and PI585348 represent the high carotenoid group. Differences in gene expressions were assessed at the group level (High vs. Low), not at the genotype level

Four additional genes in the MEP pathway were differentially expressed between high and low carotenoid lines at 42 DAP that were not differentially expressed at previous time points. Sobic.004G281900 (2-C-methyl-d-erythritol 2,4-cyclodiphosphate synthase, *ispF/MDS*) and Sobic.003G381900 (*ispD*) were more highly expressed in the high carotenoid group, while Sobic.004G207400 (*1-Hydroxy-2-methyl-2-(E)-butenyl-4-diphosphate synthase*, *ispG*) and Sobic.002G064500 (*DXS*) were expressed slightly more in low carotenoid lines (Fig. 7A, Additional File 2: Table S12). Surprisingly, 42 DAP was the only developmental stage in which carotenoid biosynthesis genes—Sobic.002G292600 (*PSY*) and Sobic.002G383400 (*PDS*)— were differentially expressed between the high versus low carotenoid lines (Figs. 7B and 8). Both *PSY* and *PDS* were more highly expressed in the high carotenoid lines. The *PSY* expression was more similar between the accessions, with the exception of PI585348 (Fig. 8A), while *PDS* had bigger differences of expressions (Fig. 8B). Among the carotenoid degradation genes, Sobic.002G225400 (*AAO*) was more highly expressed in the high carotenoid group (Fig. 7C), as it was at 14 DAP (Fig. 5B).

### Correlations between candidate gene expression and carotenoid concentration

Since we found differential expression of several *a priori* candidate genes across time points and between high vs. low carotenoid content groups, we wanted to further test the hypothesis that gene expression differences underlie carotenoid variation. To look for evidence that expression of individual *a priori* candidate genes underlie differences in individual carotenoid compounds, correlations were calculated between transcript counts and the concentrations of lutein, zeaxanthin, and β-carotene at grain maturity. Among the 55 candidate genes expressed in the grain, only 12 of them were significantly correlated (*P* < 0.05) with concentrations of at least one of the carotenoid compounds (Table 2). Expression of three genes—Sobic.003G381900 (*CDP-ME transferase*, *ispD*), Sobic.001G509200 (*CCD*), and Sobic.006G170300 (*CCD*)—were correlated with all three carotenoids (Table 2). As expected, due to their biosynthesis function in the carotenoid precursor MEP pathways, expression of *ispD* was positively correlated with concentrations of all three carotenoids. Also as expected, due to the role of CCDs in carotenoid degradation, the expression of one CCD (Sobic.001G509200) was negatively correlated with concentrations of all three carotenoids. Unexpectedly, however, expression of another CCD (Sobic.006G170300) was positively correlated with concentrations of all three carotenoids.

The expression of six genes—Sobic.009G137700 (*IPP isomerase*, *IDI*), Sobic.004G287300 (*geranyl geranyl diphosphate synthase*, *GGPPS*), Sobic.002G383400 (*PDS*), Sobic.006G232600 (*PDS*), Sobic.001G062600 (*AAO*), and Sobic.001G155300 (*NCED*)—were correlated with β-carotene and lutein concentrations, but not with zeaxanthin concentrations (Table 2). Of the MEP pathway genes, *IDI* expression was negatively correlated and *GGPPS* expression was positively correlated with β-carotene and lutein concentrations. Of the carotenoid biosynthesis genes, expression of one of the *PDS* genes (Sobic.002G383400) was positively correlated with β-carotene and lutein concentrations. However, expression of the other *PDS* gene (Sobic.006G232600) was negatively correlated with β-carotene and lutein concentrations. Of the carotenoid degradation genes, *AAO* (Sobic.001G062600) expression was positively correlated and *NCED* (Sobic.001G155300) expression was negatively correlated with β-carotene and lutein concentrations (Table 2).

Expression of three genes—Sobic.003G327200 (*CDP-ME kinase*, *ispE*), Sobic.006G177400 *(PDS)*, and Sobic.002G043500 *(AAO3)*—were correlated only with zeaxanthin concentrations. In the MEP pathway, expression of *ispE* was positively correlated with zeaxanthin concentrations. In the carotenoid biosynthesis and degradation pathways, expression of *PDS* and *AAO3* were negatively correlated with zeaxanthin concentrations, respectively (Table 2).

**Table 2:** Correlations between *a priori* candidate gene expression and concentrations of lutein, zeaxanthin, and β-carotene at 42 DAP

Transcript	Enzyme	Compound	r	p-val	Pathway
Sobic.003G381900.1	ispD	Lutein	0.960	0.040	Carotenoid precursor (MEP)
		Zeaxanthin	0.998	0.002	Carotenoid precursor (MEP)
		β-carotene	0.956	0.044	Carotenoid precursor (MEP)
Sobic.001G509200.1	CCD	Lutein	-0.990	0.010	Carotenoid degradation
		Zeaxanthin	-0.953	0.047	Carotenoid degradation
		β-carotene	-0.990	0.010	Carotenoid degradation
Sobic.006G170300.1	CCD	Lutein	0.968	0.032	Carotenoid degradation
		Zeaxanthin	0.996	0.004	Carotenoid degradation
		β-carotene	0.966	0.034	Carotenoid degradation
Sobic.009G137700.1	IDI	Lutein	-0.965	0.035	Carotenoid precursor (MEP)
		β-carotene	-0.966	0.034	Carotenoid precursor (MEP)
Sobic.004G287300.1	GGPPS	Lutein	0.951	0.049	Carotenoid precursor (MEP)
		β-carotene	0.952	0.048	Carotenoid precursor (MEP)
Sobic.002G383400.1	PDS	Lutein	0.971	0.029	Carotenoid biosynthesis
		β-carotene	0.973	0.027	Carotenoid biosynthesis
Sobic.006G232600.1	PDS	Lutein	-0.980	0.020	Carotenoid biosynthesis
		β-carotene	-0.981	0.019	Carotenoid biosynthesis
Sobic.001G062600.3	AAO	Lutein	0.969	0.031	Carotenoid degradation
		β-carotene	0.966	0.034	Carotenoid degradation
Sobic.001G155300.1	NCED	Lutein	-0.976	0.024	Carotenoid degradation
		β-carotene	-0.975	0.025	Carotenoid degradation
Sobic.003G327200.1	ispE	Zeaxanthin	0.991	0.009	Carotenoid precursor (MEP)
Sobic.006G177400.1	PDS	Zeaxanthin	-0.959	0.041	Carotenoid biosynthesis
Sobic.002G043500.1	AAO3	Zeaxanthin	-0.979	0.021	Carotenoid Degradation

### GO enrichment of differentially expressed genes for carotenoid content group

To better understand the biological role of genes differentially expressed between carotenoid content groups (High vs. Low) in each developmental stage (14, 28, and 42 DAP), a GO enrichment analysis was performed. A GO enrichment analysis is a way to characterize the function of differentially expressed genes. Genes are grouped into three main categories based on their function: biological processes (biological activity of gene product), molecular function (biochemical activity of gene product), or cellular components (location of active gene product) [36]. Within each of the three categories of GO, gene roles are further categorized into GO terms. Each GO term has a unique identifier and a name that describes the role of that term (e.g. GO:0009966, regulation of signal transduction). GO terms that are “significantly enriched” contain more genes than expected by chance, so in our data the GO terms identified as significantly enriched provide insights into processes underlying sorghum grain carotenoid variation. If a gene is not assigned a GO term, then its role is unknown.

At 14 DAP, 1,100 out of the 2,587 genes differentially expressed between the carotenoid content groups (High vs. Low) were assigned a GO annotation (Additional File 2: Table S13). Thirteen GO terms were found to be significantly enriched (FDR < 0.05), four for molecular function and nine for biological process (Additional File 1: Fig. S4, Additional File 2: Table S14). The most significant GO term at 14 DAP for molecular function was GO:0045735 (nutrient reservoir activity). Notably, all twelve genes assigned to the nutrient reservoir activity GO term encode zein proteins. For biological process, there were four highly significant GO terms: GO:0009966 (regulation of signal transduction), GO:0023051 (regulation of signaling), GO:0010646 (regulation of cell communication), and GO:0044699 (single-organism process).

At 28 DAP, 2,313 out of the 4,919 genes differentially expressed between the carotenoid content groups (High vs. Low) were assigned a GO annotation (Additional File 2: Table S13). Based on FDR < 0.05, 67 GO terms were significantly enriched for biological processes, molecular function, or cellular components at 28 DAP, which was the highest number of GO terms among the developmental stages (Additional File 1: Fig. S5, S6, and S7, Additional File 2: Table S14). The cellular components GO domain was the most enriched, with 41 significantly enriched GO terms, followed by molecular function with 17 significant GO terms, and biological processes with 9 significant GO terms (Additional File 1: Fig. S5). For cellular components, there were four highly significant GO terms: GO:0005623 (cell), GO:0044464 (cell part), GO:0005622 (intracellular), and GO:0044424 (intracellular part). Similarly, for molecular function there were four highly significant GO terms: GO:0016818 (hydroxylase activity acting on acid anhydrides), GO:0016817 (hydroxylase activity acting on acid anhydrides and phosphorous containing anhydrides), GO:0016667 (oxidoreductase activity acting on sulfur group of donors), and GO:0005198 (structural molecule activity) (Additional File 1: Fig. S6). For biological processes, three GO terms were most significant: GO:0042592 (homeostatic process), GO:0065008 (regulation of biological quality), and GO:0045454 (cell redox homeostasis) (Additional File 1: Fig. S7).

For 42 DAP, 2,109 out of 4,294 genes differentially expressed between the carotenoid content groups (High vs. Low) were assigned a GO annotation (Additional File 2: Table S13). Three GO terms were found to be enriched among the differentially expressed genes corresponding to molecular function and cellular component GO domains (Additional File 1: Fig. S8, Additional File 2: Table S14). The most significant was GO:0016491 (oxidoreductase activity) under the molecular function domain. Notably, genes annotated as flavonoid hydroxylases and cytochrome P450 enzymes were assigned to the significant GO terms.

## Discussion

Sorghum is an important staple crop in regions where vitamin A deficiency is prevalent. Although pro-vitamin A carotenoids do accumulate in sorghum grain, concentrations are low. Breeding has the potential to increase carotenoid concentrations further, but there are current gaps in knowledge of the biosynthesis and regulation of carotenoids in sorghum grain. Identifying genes that control carotenoid accumulation through grain development can help identify potential targets for biofortification breeding. Given the pattern of accumulation of carotenoids in sorghum grain through development and the large proportion of the transcriptional variation for carotenoid-related genes explained by developmental stage (Fig. 1), there seems to be tight transcriptional controls throughout grain development.

This is a promising result for biofortification breeding because it suggests that allelic variants that modify the expression patterns of the genes involved in this transcriptional switch through grain development can be used to further accumulate carotenoids in sorghum grain. For example, in maize the *crtRB1* gene, which encodes a β-carotene hydroxylase (Harjes et al., 2008; Yan et al., 2010), is differentially expressed through grain development and its expression is correlated with carotenoid content [36]. Characterization of this gene showed that there are allelic variants that result in significant differences in gene expression as well as in pro-vitamin A carotenoid accumulation [30]. Marker-assisted selection targeting the high-carotenoid allele of the *crtRB1* gene has been employed in several studies, resulting in significant increases of β-carotene concentrations [37–41]. In this study, we identified genes in the carotenoid precursor MEP pathway, carotenoid biosynthesis pathway, and carotenoid degradation pathways that have the potential to be impactful for biofortification efforts if favorable allelic variants are found.

### Carotenoid precursor pathway targets for biofortification

The MEP pathway synthesizes the GGPP precursor needed for carotenoid biosynthesis, thus the pathway is potentially involved in carotenoid variation. Several other important plant compounds, such as chlorophylls, tocochromanols, and gibberellins, also use GGPP as a precursor, so there may be competition between the pathways for use of this substrate. Identifying rate limiting steps in the MEP pathway can help to increase carotenoid concentrations in sorghum grain by increasing precursor substrates. In this study, most of the genes differentially expressed between the high vs. low carotenoid content groups were in the MEP pathway, suggesting that variation in MEP pathway genes is a major contributor to variation in carotenoid concentrations in sorghum grain. A promising MEP pathway gene target is Sobic.003G381900 (*ispD*), because it was differentially expressed across developmental time points (Fig. 2B), positively correlated with all carotenoid concentrations (Table 2), and more highly expressed in high carotenoid lines at 42 DAP (Fig. 7A). These results suggest that high expression of *ispD* increases carotenoid concentrations, perhaps by increasing the amount of precursor substrates available for the carotenoid pathway.

Sobic.004G281900 (*ispF/MDS*) is another potential MEP pathway target for biofortification breeding. Although it was not correlated with carotenoid concentrations at 42 DAP, it was more highly expressed in the high carotenoid group at 42 DAP. Notably, we previously identified this candidate gene in a GWAS for sorghum grain zeaxanthin, in which a significant marker-trait association was identified only 62 megabases away from the *ispF/MDS* gene [3]. In the same study, we also identified *ispF/MDS* in a biosynthesis-pathway targeted GWAS, which we conducted to control for possible false negatives by using only SNPs near *a priori* candidate genes. It was one of only two gene candidates identified in the pathway targeted GWAS.

Another promising MEP pathway gene target for biofortification breeding is Sobic.009G137700 (*IDI)*. It was negatively associated with carotenoid concentration for lutein and β-carotene (Table 2) and had significantly higher expression in low carotenoid lines at each developmental time point (Figs. 5A, 6 and 7 A). *IDI* catalyzes the interconversion of IPP and DMAP, the 5-carbon isomers that are condensed to form GGPP. Three molecules of IPP are sequentially added to one molecule of DMAP to form the 20 carbon GGPP, so a higher ratio of IPP to DMAP is required for sufficient chain elongation to GGPP. It could be hypothesized that the low carotenoid lines have an *IDI* allele that preferentially converts IPP to DMAPP, reducing the number of IPP molecules available for building GGPP molecules, thus increasing substrate competition for the precursors required in downstream biosynthesis pathways.

Lastly, Sobic.004G287300 (*GGPPS*) is also a promising target, as it was differentially expressed across developmental time points (Fig. 2), associated with lutein and β-carotene (Table 2), and differentially expressed between high and low carotenoid groups for all developmental time points (Figs. 5A, 6 and 7 A). The expression pattern of this *GGPPS* gene is perhaps the most exciting in the MEP pathway, because it shows differential expression between high and low carotenoid content groups for all developmental time points. *GGPPS* is highly expressed in both high and low carotenoid groups at 14 DAP (Fig. 2B), however, at 28 DAP and 42 DAP, expression is decreased for the low carotenoid group, but remains high in the high carotenoid group (Figs. 6 and 7 A, Additional File 1: Fig. S1A, S2A and S3A). GGPPS catalyzes the third condensation of IPP with DMAPP to form GGPP. We hypothesize that high carotenoid lines have an allelic variant that remains highly expressed through late stages of grain development, thus increasing the flux to the carotenoid biosynthesis pathway. In maize, a positive correlation between carotenoid concentration and gene expression of *GGPPS* has also been detected [29]. We previously identified a different sorghum *GGPPS* homolog (Sobic.002G353300) in a β-carotene genome-wide association study, but the sorghum homolog differentially expressed in this study (Sobic.004G287300) has not previously been associated with variation in carotenoid content [3].

### Carotenoid biosynthesis pathway targets for biofortification

Genes directly involved in the carotenoid biosynthesis pathway have been the major targets for biofortification efforts across species due to their potential to control specific carotenoids, such as β-carotene. In this study two carotenoid pathway genes were identified as promising targets for biofortification efforts. The first gene, Sobic.002G383400, encodes a PDS that was positively correlated with lutein and β-carotene concentrations (Table 2), was more highly expressed at late stages of grain development, and was more highly expressed in the high carotenoid lines versus the low carotenoid lines at 42 DAP. One hypothesis is that the increase in *PDS* expression at late stages of development in the high carotenoid group increases conversion of phytoene to phytofluene (9,15,9’-tri-cis-**ζ**-carotene), the second carotenoid in the biosynthesis pathway, thus increasing all subsequent carotenoids in the pathway (Fig. 3). However, this gene has a low transcript count number when compared to other biosynthesis genes (Figs. 7B and 8B), so further characterization is required to understand the role of *PDS* in carotenoid variation. Genetic differences in transcript abundance may not reflect genetic variation in enzyme abundance, since there may be additional variation in post-transcriptional regulation.

The second promising gene is Sobic.002G292600 (*PSY)*. This gene is an interesting candidate because, unlike the other candidates, it was not differentially expressed across time points (Fig. 3), nor was it correlated with carotenoid concentrations (Table 2), but it was differentially expressed between high vs. low carotenoid content groups at 42 DAP. Interestingly, our highest β-carotene content line, PI585348, had notably higher *PSY* expression compared with the other accessions at 42 DAP (Fig. 8A). In a QTL mapping study in a biparental sorghum population, SNPs in proximity to this gene were found to be associated with lutein, zeaxanthin, and β-carotene concentrations [4], suggesting there are *PSY* allelic variants associated with variation in β-carotene concentration. *PSY* genes have also been associated with carotenoid concentrations in maize [35], as well as with differential expression through development and between genotypes [7, 23, 28, 36, 42]. As the first committed step in carotenoid biosynthesis, increasing PSY activity could potentially increase carotenoid accumulation in sorghum grain. Sequencing this gene in PI585348 will help to identify potential allelic variants responsible for the high expression and high carotenoid content.

It is worth noting that the *ZEP* gene (Sobic.006G097500) was not differentially expressed in carotenoid content groups (High vs. Low) even though it was identified as a major GWAS candidate in a previous study [3] and ZEP differential expression has been found to underlie carotenoid variation in maize and Arabidopsis [29, 43]. These results suggest that ZEP differential expression does not underlie the association between the *ZEP* region and carotenoid variation. It is possible that the marker-trait association identified in our GWAS study is in linkage disequilibrium with *ZEP*, but that *ZEP* is not the functional variant. However, this is unlikely because there is significant evidence in maize and arabidopsis that *ZEP* is one of the major controllers of grain carotenoid variation [22, 24, 26, 27]. One hypothesis is that there are allelic variants of *ZEP* that affect enzyme or transcript stability rather than gene expression. Sequencing of the *ZEP* gene and measuring enzymatic activity in accessions with diverse carotenoid profiles is needed to test this hypothesis.

### Carotenoid degradation pathway targets for biofortification

Degradation of carotenoids occurs throughout the biosynthesis pathway. Two main routes of degradation are degradation of β-carotene to apocarotenoids such as strigolactones, or degradation of violaxanthin and neoxanthin to ABA (Fig. 4). Three enzyme groups—CCDs, NCEDs, and AAOs—catalyze the degradation steps (Fig. 4). Almost all of the *a priori* candidate genes were differentially expressed through grain development (Fig. 4B C). However, in the high vs. low carotenoid content groups, only five degradation genes were differentially expressed at each time point (Figs. 5B and 6B, and 7 C).

Two of the differentially expressed degradation genes—Sobic.001G509200 (*CCD*) and Sobic.001G155300 (*NCED*)—were not differentially expressed among the high vs. low carotenoid content groups, but were differentially expressed through development, with both of them more highly expressed in later time points (Fig. 4C). A QTL mapping study in sorghum identified QTLs in proximity to both of these genes that were associated with lutein variation [4], suggesting there may be allelic variants of these two genes associated with higher carotenoid concentrations. Allelic variants with reduced activity in either of these genes could be used in molecular breeding to reduce degradation of carotenoids at later stages of grain development. However, despite their potential, carotenoid cleavage enzymes are known to have low substrate specificity [44–46], thus they could have limited impact if complementary action of other degradation enzymes occurs.

Surprisingly, there was a positive correlation between carotenoid concentrations and Sobic.006G170300, annotated as a CCD (Table 2). CCD’s are a family of enzymes known to degrade some carotenoids to apocarotenoids, which are a large diverse group of compounds—including ABA and strigolactones—derived from carotenoids through oxidative cleavage. Therefore we expect negative correlations between CCD’s and carotenoid concentrations. In Arabidopsis and maize grains, negative correlations have been observed between *CCD* expression and carotenoid content [7, 47]. The four major CCD’s in plants—CCD1, CCD4, CCD7, and CCD8—share specificity for the 9,10 double bonds on their substrates, and have been shown to be tissue specific. *CCD7* and *CCD8* have tissue specificity for Arabidopsis roots [47], while *CCD1* and *CCD4* have been found to be expressed in fruits and flowers of several plants [45]. Based on sequence similarity with *Arabidopsis thaliana*, Sobic.006G170300 encodes the CCD7 (74.4% similarity to AT2G44990). Even though Sobic.006G170300 overcame our 10 count filtering threshold, it had a low expression level with only 25 counts across our samples (Supplementary Table 2). Therefore it may be possible that the positive correlation here observed (Table 2) is a false positive due to low counts and that Sobic.006G170300 encodes a CCD7 with predominant root expression. To test this hypothesis, sorghum CCD’s across tissues must be examined. Alternatively, we could hypothesize that the Sobic.006G170300 allele in the high carotenoid lines encodes an enzyme that has reduced carotenoid degradation activity. To test this hypothesis, the gene can be sequenced in both the high and low carotenoid lines for comparison, and enzyme activity can be measured and compared.

### Duplicate genes and their potential for biofortification

Interestingly, we identified several duplicate genes that appear to be complementary to each other, with one duplicate expressed only in early grain development and the other expressed only in late grain development. For example, PDS genes Sobic.006G177400 and Sobic.002G383400 were more highly expressed at early stages of development and at later stages of development, respectively. We hypothesize that this expression divergence is due to subfunctionalization (the expression patterns of the ancestral gene is divided between the duplicates, so the ancestral expression pattern is not retained) or neofunctionalization (one duplicate gains a new expression pattern and the other duplicate retains the ancestral expression pattern) [48]. The type of expression divergence in duplicate genes has implications for biofortification breeding, because the way in which duplicate genes function together will inform the choice in breeding strategies. For example, if increased expression of a duplicate gene is needed to increase carotenoid concentrations, and the duplicate genes have redundant function, then only one gene may need to be manipulated for biofortification. In contrast, if decreased expression of a gene is needed to increase carotenoid concentrations, then both genes may need to be manipulated for effective biofortification. However, if the duplicate genes do not have redundant function, then manipulating any one of them for biofortification may result in unintended effects due to pleiotropy. Gene complementation tests in recombinant inbred lines (RILs) could be used to test the relationships between these duplicated genes.

### Carotenoid regulation in sorghum

Genomic studies suggest that variation in sorghum grain carotenoids are due to an oligogenic genetic architecture, with marker-trait associations detected near *a priori* candidate genes within the carotenoid precursor MEP pathways, the carotenoid biosynthesis pathway, and the carotenoid degradation pathways [3, 4]. However, only one of the genes (*ispF*/*MDS*) identified in previous GWAS was here identified as differentially expressed in high vs. low carotenoid content groups. Comparing these results with those of maize [7, 24, 28, 29, 37], in which several GWAS-identified gene candidates were found to be differentially expressed between high and low carotenoid lines, it seems that the controls for sorghum carotenoid content may be different. One hypothesis is that the genetic variation underlying carotenoid variation in sorghum is not causing significant differences at the transcriptional level. Alternatively, given the interconnectivity of carotenoid biosynthesis with other biological processes, carotenoid content in sorghum grain could have a more complicated inheritance and control. The significant enrichment of non-carotenoid GO terms suggest this may be the case.

As secondary metabolites, carotenoids interact with multiple biochemical pathways across tissues and cellular compartments, and they involve many molecular functions as shown in our GO enrichment analysis (Supplemental Fig.s 4, 5, 6, 7, and 8). For example, identification of the twelve zein genes associated with the nutrient reservoir GO term at 14 DAP suggest the possibility that the major endosperm storage proteins may interact with carotenoids and have a role in carotenoid accumulation during grain fill. This possibility was discussed in a recent maize study in which *β-OH*, named *ven1* in the study, was found to modulate kernel texture [49]. Furthermore, identification of the flavonoid hydroxylase genes associated with oxidoreductase activity at 42 DAP suggest possible interactions with other biochemical pathways. Four of the flavonoid hydroxylase genes are orthologs or homologs of the arabidopsis *TT7*, a key enzyme in the flavonoid biosynthesis pathway. One hypothesis is that there may be interactions between the two biosynthesis pathways that influence the accumulation of carotenoids and flavonoids. Few studies have investigated this, but a recent study in navel oranges (*Citrus cinencis*) identified potential co-regulators of carotenoids and flavonoids [50]. An alternative hypothesis is that the hydroxylase genes, which are cytochrome P450 enzymes, are functioning in the carotenoid pathway independent of the flavonoid pathway. Cytochrome P450 enzymes are known to catalyze hydroxylations in the carotenoid pathway, so the hydroxylases identified in the GO analysis may have a yet unidentified role in the sorghum carotenoid pathway. An overarching hypothesis is that at the gene expression level, although many of the differentially expressed genes do not independently have large expression differences between high versus low carotenoid content groups, the additive effect of their expression results in higher carotenoid concentrations, suggesting a polygenic component of carotenoid inheritance in sorghum grain.

## Conclusions

To gain insights into the mechanisms underlying sorghum carotenoid biosynthesis and variation, we used a functional genomics approach, analyzing the transcriptomes in high and low carotenoid sorghum varieties throughout grain development. We have developed a clearer understanding of carotenoid regulation in sorghum grain, finding that (1) early MEP and carotenoid biosynthesis pathway genes are more highly expressed during early grain development, whereas later MEP and carotenoid biosynthesis pathway genes are more highly expressed during late grain development; (2) there is differential expression between high and low carotenoid lines of predominantly MEP and carotenoid degradation pathway genes during early grain development, whereas there is differential expression of genes in all three pathways during late grain development; and (3) controls for sorghum grain carotenoids are likely organized across a metabolic network, interacting with pathways involved in synthesizing other compounds during grain fill. Additionally, we identified potential gene targets for biofortification breeding, particularly *GGPPS*, *PSY*, and *PDS*. Moving forward, QTL analysis, sequence analysis, and marker testing are needed to identify causal allelic variants underlying carotenoid variation in sorghum. This transcriptomics study contributes to efforts to develop molecular breeding tools for sorghum carotenoid biofortification.

## Methods

### Plant material

Four sorghum accessions—PI329435, PI510924, PI585347, and PI585348—with contrasting carotenoid profiles (Additional File 2: Table S1) were grown in the Plant Growth Facilities greenhouse of Colorado State University from March 2021 to July 2021. Accessions were planted using a complete randomized design (CRD) with nine replicates per accession. Grain was collected at three time points 14, 28, and 42 days after pollination (DAP), here defined as the first day pollen emerged on the top part of the panicle. These timepoints were selected because in our germplasm it corresponds with the milky, soft dough and hard dough developmental stages therefore tissue was collected at the same physiological stage for each accession. For each accession, 3 biological replicates of each time point were collected. Grain samples were immediately flash frozen with liquid nitrogen and stored at -80 C until RNA extractions.

### RNA extraction, libraries and sequencing

Total RNA was extracted from grain samples using a SDS-LiCl method [51] with some modifications. Briefly, 500 ul of chilled extraction buffer (100mM Tris-HCL (pH = 8), 25 mM EDTA 2Na, 2.5% PVP, 2.5 M NaCl, 2.5% β-Mercaptoethanol in DEPC-water), 2 steel grinding balls, and 6 sorghum seeds were added to 2mL tubes. Next, they were immediately transferred to a Bead Ruptor Elite (Omni International, Kennesaw, GA) and ground for 30 s at a speed of 4 m/s. Samples were transferred to ice and an additional 500 µl of chilled extraction buffer was added to each sample and mixed with a vortexer. The homogenate was incubated for 5 min at room temperature and 100 µl of 20% SDS was added to the suspension. Then, samples were incubated at room temperature for 2 min and centrifuged at 16,000xg for 5 min at 4℃. The supernatant was collected and 800 µl of phenol:chloroform:isoamyl alcohol (25:24:1) was added. Then, the homogenate was mixed with a vortexer and centrifuged at 16,000xg for 5 min at 4 °C. After centrifugation, the upper aqueous phase was collected and 200 µl of chloroform was added. Samples were then vortexed and centrifuged at 16,000×g for 5 min at 4 °C. The upper aqueous phase was collected after centrifugation then, 160 µl of 8 M LiCl and 50 µl of 3 M sodium acetate (pH 4.8) were added and mixed by gentle inversion. To precipitate RNA, samples were then incubated at -20℃ for 24 h. After precipitation, the samples were centrifuged at 16,000×g for 5 min at 4 ℃. Following centrifugation, the pellet was kept and 500 µl of 2 M LiCl was added and mixed gently. Samples were then centrifuged at 16,000×g for 5 min at 4 ℃ and the supernatant was discarded. To clean the pellet, 500 µl of pre-chilled 80% ethanol was added and mixed by inversions followed by centrifugation at 16,000×g for 5 min at 4 ℃. The pellet was dried using a speed vac and re-suspended in 30 µl of DEPC-water. RNA was then stored at -80 ℃ for downstream applications.

### RNA sequencing, alignment, and transcript counts

Total RNA obtained was sent to the Kansas State University Integrated Genomics Facility (IGF) for library preparation and sequencing. The 36 samples (Additional File 2: Table S1) were pooled into three libraries and sequenced independently. Single end-paired reads were obtained from NextSeq 500 with the NextSeq 500/550 High Output v2.5 Kit (75-nucleotide single-end reads) (Illumina, US). The obtained reads were aligned to the sorghum reference genome v3.1.1 using the splice-aware aligner GMAP/GSNAP program version “2021-12-17” [52]. The aligned transcripts were then counted using HTSeq-counts [53] (Additional File 2: Table S2). Genes with transcript count numbers of 10 or more were kept for subsequent analysis.

### A priori candidate genes

Given the extensive knowledge of carotenoid biosynthesis, analysis was focused on *a priori* candidate genes. Transcript information for genes involved in the carotenoid biosynthesis pathway, carotenoid degradation, and the carotenoid precursor MEP pathway were obtained from Phytozome (http://www.phytozome.net) and Kyoto Encyclopedia of Genes and Genomes (KEGG, https://www.genome.jp/kegg) (Additional File 2: Table S3). Several candidate genes had more than one transcript expressed, therefore, ‘genes’ is used when referring to all of the transcripts and ‘transcript’ when referring only to the specified transcript. Note that since genes in the carotenoid pathway have been identified in many species, there are multiple names for most of them, some of which are compiled in Additional File 2: Table S3. This is particularly true for the *β-carotene 3-hydroxylase* gene. We chose to use the notation *β-OH*, but names used in other papers include *crtZ*, *crtRB1*, *BCH*, and *hyd3*.

### Clustering of samples

Transcript counts were transformed with the “varianceStabilizingTransformation” and transformed data was obtained with the “getVarianceStabilizedData” function of the R package DESeq2 [54], using developmental time points as the design variable. The transformed counts were transposed and a Euclidean distance matrix was calculated using the “dist” R base function. Average hierarchical clustering was determined with the “hclust” function in R. Relationships of sample clustering and total carotenoid concentration in mature grains for each sample were visualized with the “plotDendoAndColors” function of the WGCNA R package [55] .

### Differentially expressed genes

Genes differentially expressed were determined using the DESeq2 package [54] in R. Genes differentially expressed across DAP were identified using the likelihood ratio test, with false discovery rate (FDR) < 0.05. Differentially expressed genes for DAP were then separated according to the biochemical pathways, and the patterns of expressions across the DAP were analyzed with the “degPatterns” function. The Wald test was used to identify genes differentially expressed in the carotenoid concentrations groups (High vs. Low). Normalized transcript counts were obtained for differentially expressed genes in the high vs. low carotenoid content groups using DESeq2 median of ratios which accounts for sequencing depth and RNA composition. Normalized counts then were aggregated by the carotenoid content group (high vs. low) for comparisons.

### Correlations between candidate genes and carotenoid concentrations

Correlations between lutein, zeaxanthin, and β-carotene were determined for each of the candidate genes in the carotenoid biosynthesis, carotenoid degradation, and the carotenoid precursor MEP pathway. Average carotenoid concentrations (µg/g) of one biological rep from previous studies in 2015 [3] and 2019 [56] were used (Additional File 2: Table S1). Transcript counts for 42 DAP were transformed with the VST transformation and averaged across replicates of the four accessions—PI510924, PI329435, PI585347, and PI585348. Pearson’s correlations between each carotenoid concentration and the transformed transcript counts for each gene were calculated in R. Candidate genes with *P*-value < 0.05 were found to be significantly correlated.

### GO enrichment analysis

Genes differentially expressed for the carotenoid concentration groups (‘High vs. Low’) for each developmental time point (14, 28, and 42 DAP) were independently selected for Gene ontology (GO) enrichment analysis. Enriched GO terms were identified using the Singular Enrichment Analysis (SEA) analysis tool in agriGO website (http://systemsbiology.cau.edu.cn/agriGOv2/index.php) [57]. *Sorghum bicolor* was selected as the species under the Poaceae Plant group analysis and GO terms were identified using the Sorghum genome version 3.1 from Phytozome v11.0 as the reference. Statistical parameters used to identify significantly enriched GO terms were the Fisher test with Hochberg multi-test False Discovery Rate adjustment, a minimum number of mapping entries of 5, and a significance level of < 0.05.

## Electronic supplementary material

Below is the link to the electronic supplementary material.

Supplementary Material 1

Supplementary Material 2

## Data Availability

The datasets generated and analyzed during the current study are available in the NCBI repository, under Bioproject: PRJNA877616 [https://www.ncbi.nlm.nih.gov/bioproject/?term=PRJNA877616 ].

## List of abbreviations

AAO: abscisic aldehyde oxidase
ABA: abscisic acid
β-OH: β-carotene 3-hydroxylase
CCD: carotenoid cleavage dioxygenases
CRTISO: prolycopene isomerase
DAHB: days after half bloom
DAP: days after pollination
DMAPP: dimethyl pyrophosphate
DXS: deoxy xylulose 5-phosphate synthase
FDR: false discovery rate
FPPS: farnesyl pyrophosphate synthase
GGPPS: geranyl geranyl pyrophosphate synthase
GO: Gene ontology
GPPS: geranyl diphosphate synthase
IDI: IPP isomerase
ispD: CDP-ME transferase
ispE: CDP-ME kinase
ispF/MDS: 2-C-methyl-d-erythritol 2,4-cyclodiphosphate synthase
ispG: 1-Hydroxy-2-methyl-2-(E)-butenyl-4-diphosphate synthase
IPP: isopentyl pyrophosphate
ME-2,4cPP: 2-C-methyl-D-erythritol 2,4-cyclodiphosphate
MEP: methylerythritol phosphate
NCED: 9-cis-epoxycarotenoid dioxygenases
PSY: phytoene synthase
PDS: phytoene desaturase
QTL: quantitative trait loci
VDE: violaxanthin de-epoxidase
VST: variance stabilizing transformation
ZEP: zeaxanthin epoxidase
