# Supplementary material for: Unraveling transcriptomics of sorghum grain carotenoids: a step forward for biofortification
Source: BMC Genomics. 2023 May 3;24:233. doi: 10.1186/s12864-023-09323-3 (PMC10157909; doi:10.1186/s12864-023-09323-3)
Supplement: Supplementary file 2 — Supplementary Material 2 [file 12864_2023_9323_MOESM2_ESM.docx]

# **Supplemental Figures**


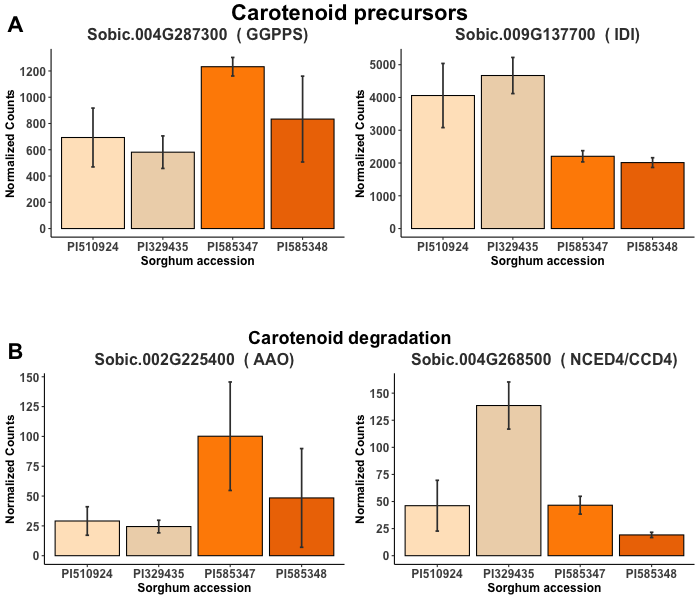

**Figure S1.** Differentially expressed genes at 14 DAP for high versus low carotenoid sorghum accessions. Genotypes normalized transcript counts for A) carotenoid precursors (MEP pathway); B) carotenoid degradation pathways


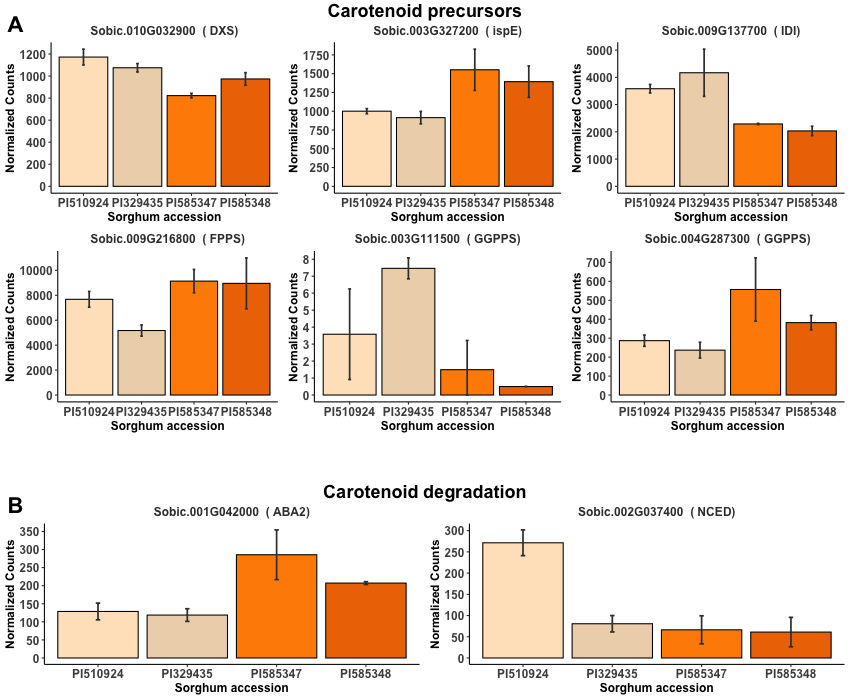


**Figure S2.** Differentially expressed genes at 28 DAP for high versus low carotenoid sorghum accessions. Genotypes normalized transcript counts for A) carotenoid precursors (MEP pathway); B) carotenoid degradation pathways


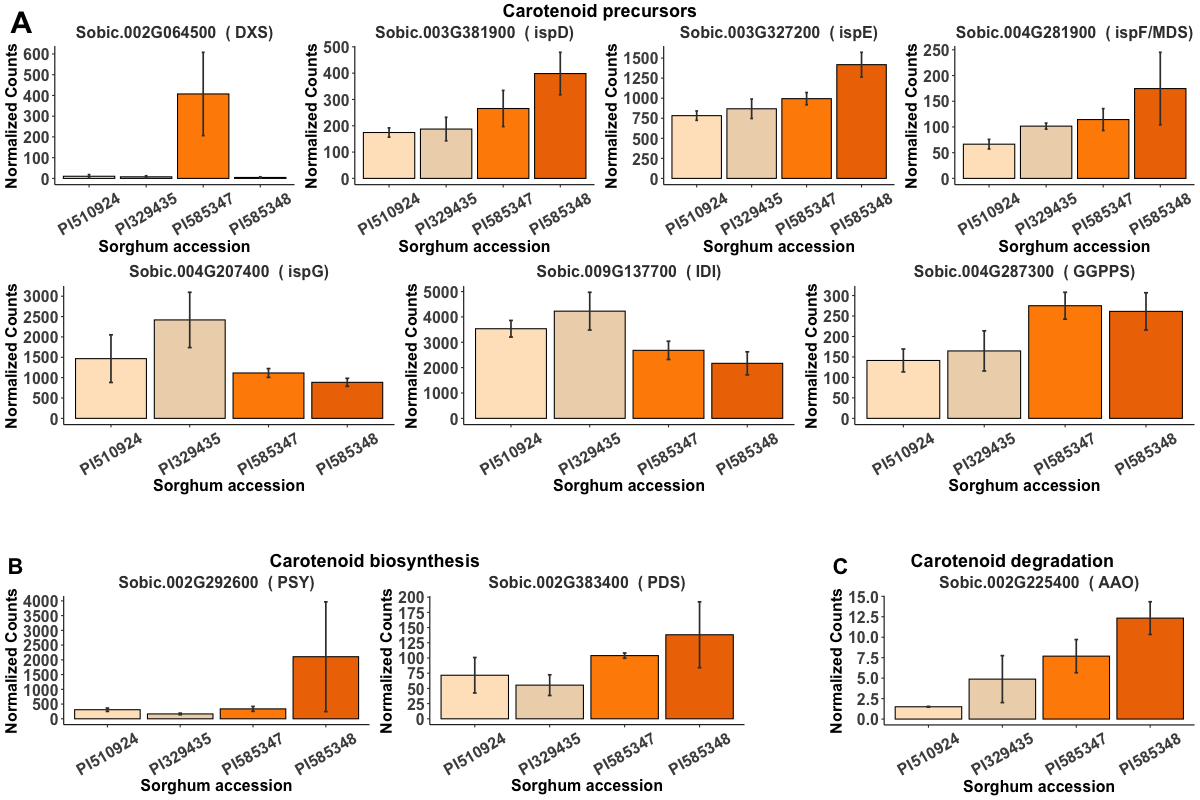


**Figure S3.** Differentially expressed genes at 42 DAP for high versus low carotenoid sorghum accessions. Genotypes normalized transcript counts for A) carotenoid precursors (MEP pathway); B) carotenoid biosynthesis; C) carotenoid degradation pathways


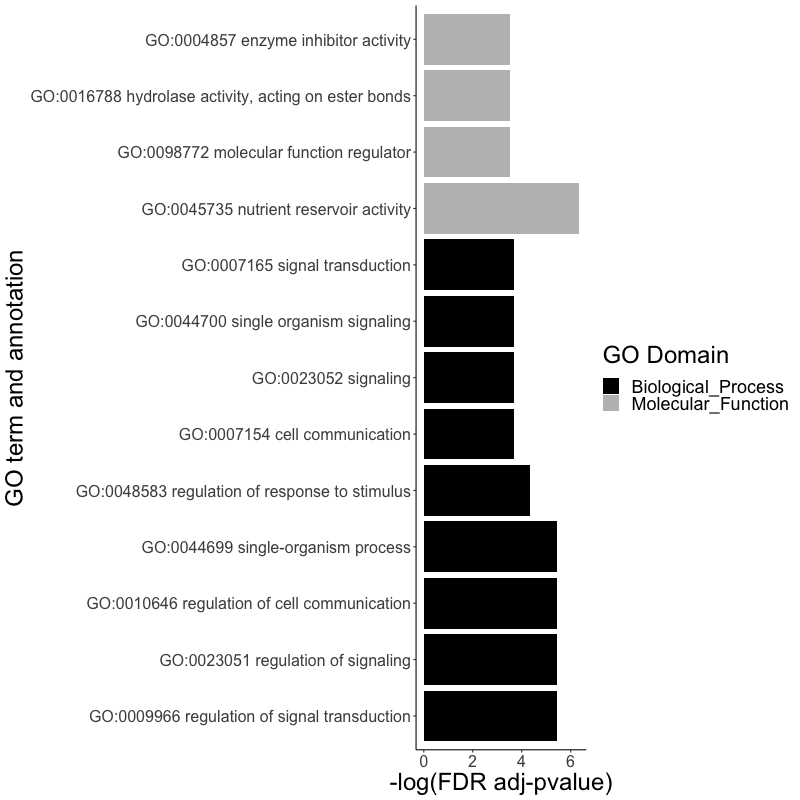


**Figure S4.** Significant GO terms in the SEA GO enrichment analysis for differentially expressed genes in carotenoid content group (High vs Low) at 14 DAP.

**
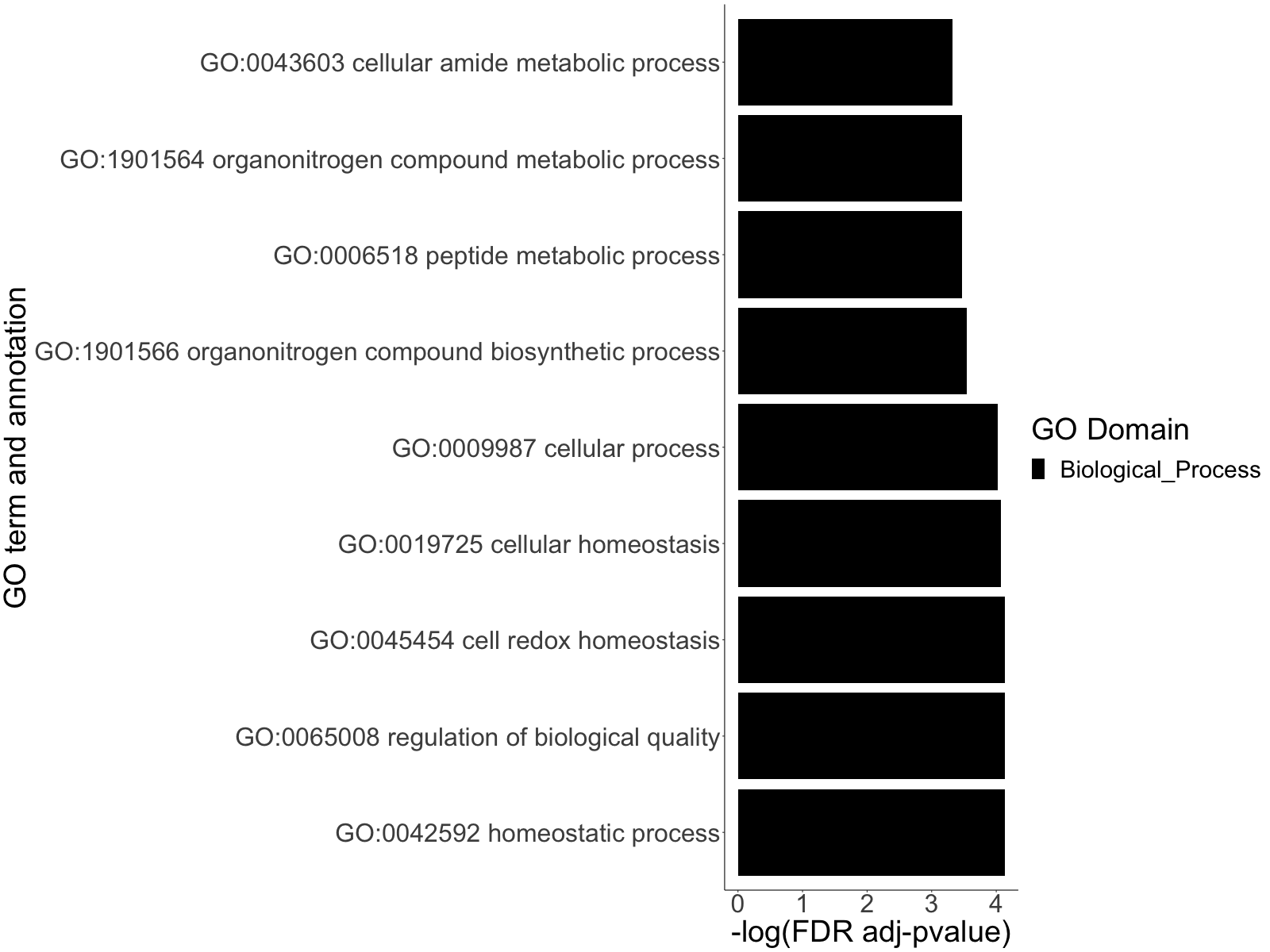
**

**Figure S5.** Significant GO terms under the biological process domain in the SEA GO enrichment analysis for differentially expressed genes in carotenoid content group (High vs Low) at 28 DAP.


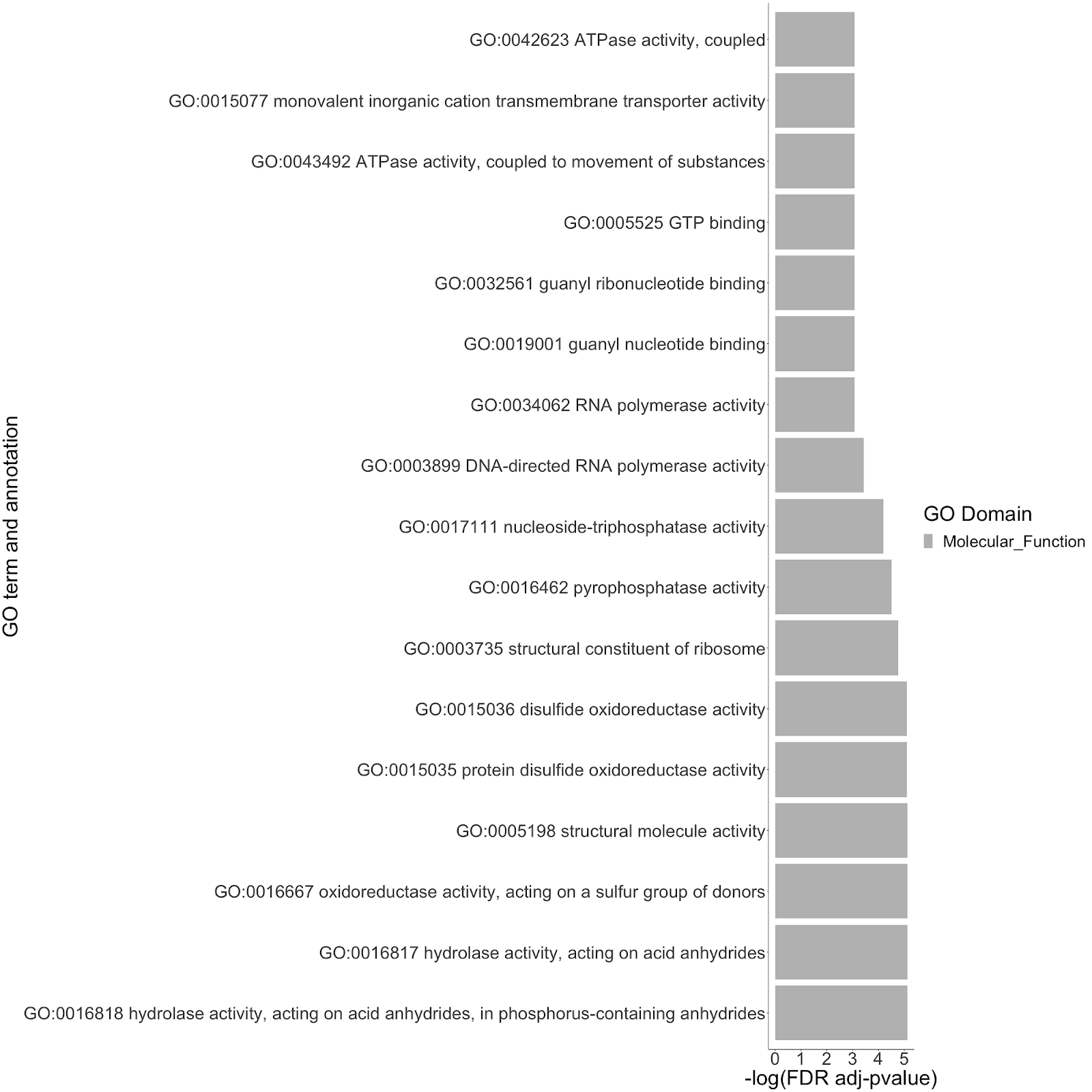


**Figure S6.** Significant GO terms under the molecular function domain in the SEA GO enrichment analysis for differentially expressed genes in carotenoid content group (High vs Low) at 28 DAP.


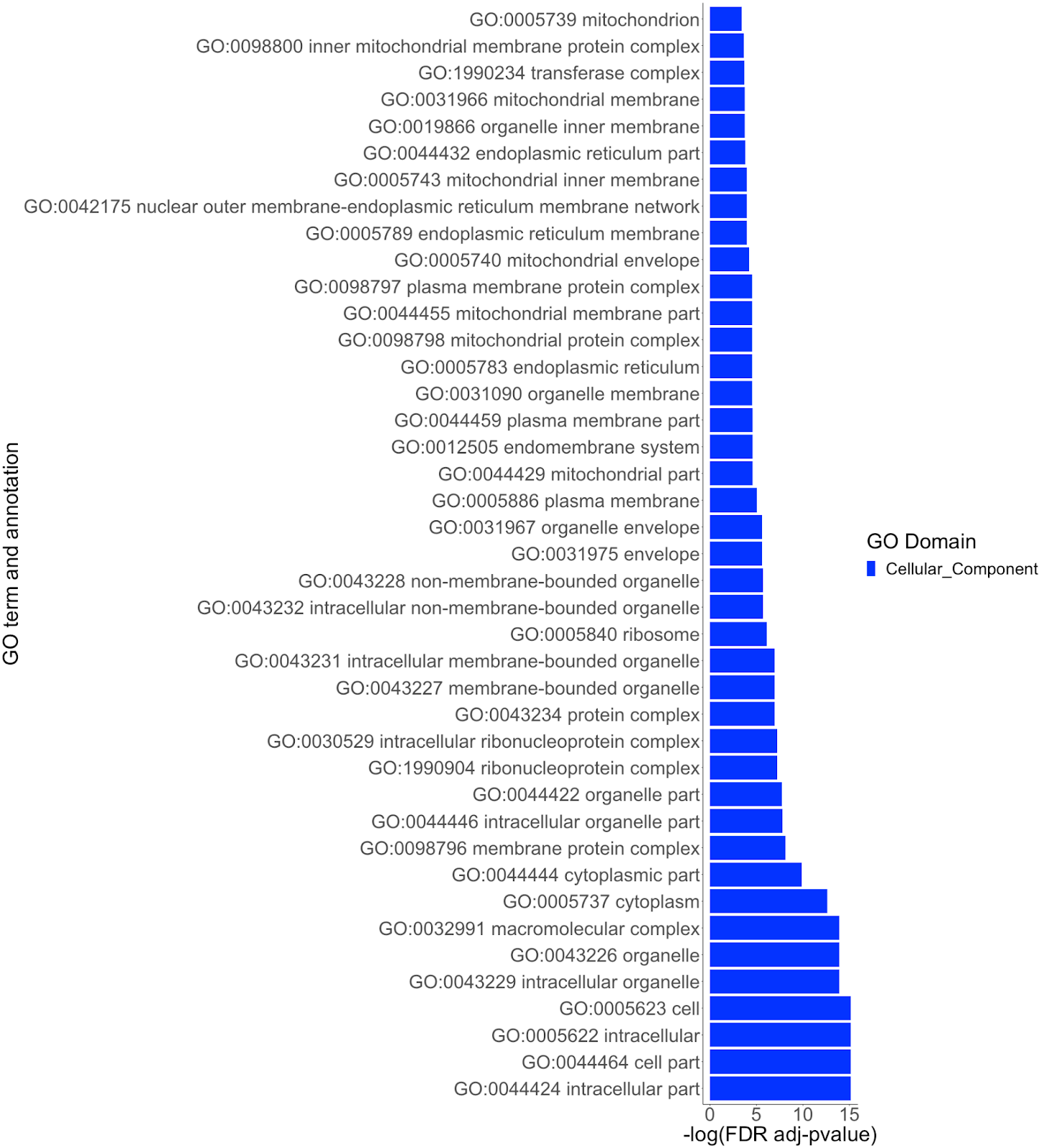


**Figure S7.** Significant GO terms under the cellular component domain in the SEA GO enrichment analysis for differentially expressed genes in carotenoid content group (High vs Low) at 28 DAP.


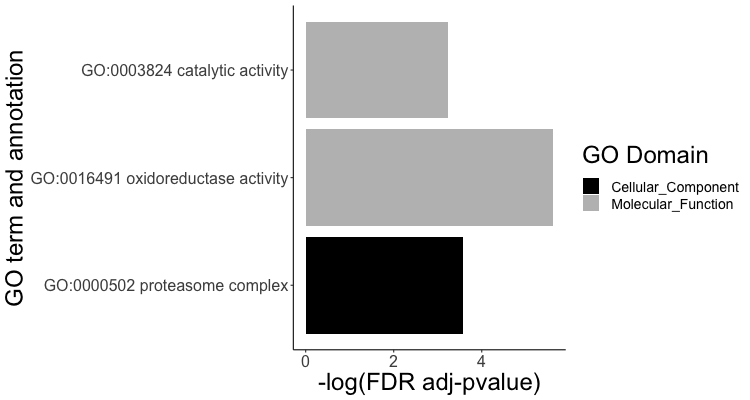


**Figure S8.** Significant GO terms in the SEA GO enrichment analysis for differentially expressed genes in carotenoid content group (High vs Low) at 42 DAP.
